# The central role of self-esteem in the quality of life of patients with mental disorders

**DOI:** 10.1038/s41598-022-11655-1

**Published:** 2022-05-12

**Authors:** Guillaume Barbalat, Julien Plasse, Emmanuel Gauthier, Hélène Verdoux, Clélia Quiles, Julien Dubreucq, Emilie Legros-Lafarge, Nematollah Jaafari, Catherine Massoubre, Nathalie Guillard-Bouhet, Frédéric Haesebaert, Nicolas Franck

**Affiliations:** 1 Centre Ressource de Réhabilitation Psychosociale et de Remédiation Cognitive, Hôpital Le Vinatier, Pôle Centre rive gauche, UMR 5229, CNRS & Claude Bernard Unversity Lyon 1, Université de Lyon, Lyon, France; 2 University of Bordeaux, Bordeaux, France; 3 Centre Hospitalier Universitaire de Saint-Étienne, Saint-Étienne, France; 4 University of Limoges, Limoges, France; 5 University of Poitiers, Poitiers, France

**Keywords:** Health services, Quality of life

## Abstract

In psychiatry, recent years have seen a change of focus from a clinician- to a patient-centered perspective that emphasizes quality of life as a treatment target. As a complex construct, quality of life is composed of multiple dimensions that interact with one-another (e.g. physical and psychological well-being, relationships, autonomy, self-esteem). Here, we used data from the REHABase cohort, which includes N = 2180 patients from 15 psychosocial rehabilitation centers in France, to explore networks of quality-of-life dimensions among six psychiatric disorders: schizophrenia, neurodevelopmental, bipolar, depressive, anxiety, and personality disorders. Stronger connections (edges) involved the Self-Esteem dimension, such as Self-Esteem–Physical Well-Being, Self-Esteem–Autonomy, Self-Esteem–Psychological Well-Being, and Self-Esteem–Resilience. Self-esteem was also consistently retrieved as the most central node (the dimension with the most connections within each network). Between-group tests did not reveal any differences regarding network structure, overall connectivity, edge-weights, and nodes’ centrality. Despite presenting with different symptom profiles, various psychiatric disorders may demonstrate similar inter-relationships among quality-of-life dimensions. In particular, self-esteem may have a crucial inter-connecting role in patients’ quality of life. Our findings could support treatment programmes that specifically target self-esteem to improve patients’ quality of life in a cost-effective way.

## Introduction

In accordance with the WHO definition of health, which stipulates that, as “a fundamental right”, “health is a state of complete physical, mental and social well-being and not merely the absence of disease or infirmity”^1^, psychiatry has seen a recent move from a clinician- to a patient-centered outcome perspective that focuses treatment goals towards quality of life and functioning rather than merely clinical signs^2^.

To date, health services research on quality of life has mostly focused on how socio-demographic and clinical factors, including the duration and intensity of symptoms, would best predict the quality of life of those with psychiatric disorders^3–12^. These analyses however are relatively restrictive because, as clinical and scientific observations have demonstrated, quality of life is not solely the product of mental symptoms, but also likely impacts clinical and socio-demographic characteristics^13,14^. Moreover, quality of life is itself a complex construct made of multiple dimensions, which encompass physical and emotional well-being, social functioning and relationships, resilience, autonomy, and self-esteem^15^. Crucially, these dimensions interact with each other^16^. For instance, a patient with depression might theoretically improve his/her psychological well-being if he/she places strong emphasis on his/her physical well-being, via the restoration of his/her self-esteem.

A full shift towards a patient-centered outcomes perspective could be enabled by understanding how quality-of-life dimensions interact; whether their connections with one-another are strong; which dimensions are specifically stronger; and whether some dimensions are overall more connected than others. Such information could be of high significance if, for instance, one dimension was to be retrieved at the core of a system of quality-of-life dimensions: one would hypothesize that focussing treatment on this specific dimension could substantially improve patients’ quality of life and functioning in a cost-effective way. Clearly, this requires another change of gears, this time from a research perspective, towards systems rather than pure analytical science. Unfortunately however, such knowledge is currently lacking.

In fact, these insights match well with a recent conceptualization of psychological problems as being the result of networks of interacting components (e.g. symptoms, behaviours, or other dimensions) rather than an underlying entity (typically, a reified psychiatric disorder)^17^. This conceptualization usually allows a more realistic and practical description of psychological suffering thereby reframed under the terminology of “psychological networks”^18,19^. Psychological networks analysis usually involves estimating a statistical model on data, which is a weighted network representing strengths of relationships between dimensions of interest. Subsequently, one would be interested in running statistical tests on these networks (within- and between-networks), for instance on their global structure, global and local connection strengths, but also by pointing out which dimensions are core to the systems of interactions.

In the current study, we aimed at using these techniques to explore networks of quality-of-life dimensions among various psychiatric disorders, specifically: schizophrenia, neurodevelopmental, bipolar, depressive, anxiety, and personality disorders. We were interested in gathering information on the global structure of each disorder’s network, as well as its overall connectivity, the strength of each connection between two dimensions, and the relative importance of each dimension. We extracted data from the REHABase cohort database^20^, which has been previously used in observational descriptive and associational studies on mental health services research^20–26^. The database contains observations across multiple psychosocial rehabilitation centres in France and presents the notable advantage of being both large and real-life.

## Methods

### Data

#### Population

Our dataset consists of patients enrolled in the REHABase cohort. Briefly, the cohort includes patients with serious mental illness referred to 15 centers of a French psychosocial rehabilitation network—described in^20^. Clinically stabilized patients are referred to the centers by public mental health services, private psychiatrists, general practitioners, or are self-referred. They undergo a standardized clinical, functional and cognitive evaluation performed by a multidisciplinary team (psychiatrists, nurses, neuropsychologists, occupational therapists and social workers) in order to subsequently benefit from a personalized rehabilitation care plan over a 1-year period (for instance, cognitive remediation, social skills training, vocational rehabilitation, etc.). A standardized electronic case report form is used to collect demographic, clinical, functioning, and cognitive data. Regular group meetings are held to monitor quality control and ensure good inter-rater reliability^20^.

Our analysis was restricted to patients included in the REHABase cohort from January 2016 to September 2021. For our main analysis, each patient presented with a DSM-5 diagnosis of schizophrenia spectrum disorder; autism spectrum disorder; bipolar disorder; depressive disorder; anxiety disorder; post-traumatic stress disorder; obsessive compulsive disorder (the latter three disorders were further regrouped under the umbrella of anxiety disorder); or personality disorder, based upon clinical interview performed by a psychiatrist^27^. Note that we also performed similar analysis without any diagnosis restrictions.

#### Quality-of-life measures

In the current study, we used data collected on the Schizophrenia Quality of Life-18 (S-QoL18)^28^, a self-administered questionnaire of 18 items, which are then regrouped under the following quality-of-life (QoL) dimensions (score 0–100; high score indicates better QoL): self-esteem (SEL), romantic life (ROM), resilience (RES), psychological well-being (PSY), physical well-being (PHY), relationships with friends (FRI), family relationships (FAM), autonomy (AUT). The SQoL-18 has many convenient properties: (1) it is a short and easy-to-use instrument; (2) it has satisfactory psychometric properties, with a high degree of comparability with a longer, well-validated version of 41 items^28^; and (3) it covers eight key aspects of quality of life.

Our initial sample consisted of 3948 patients, of whom N = 2180 were fully screened for quality of life. Details of the sample, including socio-demographic and clinical characteristics, can be found in Table 1.

**Table 1:** Clinical and socio-demographic characteristics of participants.

Variables	Schizophrenia spectrum disorders (N = 925)	Neurodvpt disorders (N = 212)	Bipolar disorders (N = 275)	Depressive disorders (N = 133)	Anxiety disorders (N = 179)	Personality disorders (N = 225)	All REHABase patients (N = 2180)
Age, mean (SD)	32.5 (9.8)	28.0 (9.4)	38.9 (10.6)	35.5 (11.9)	31.2 (10.5)	31.2 (9.5)	32.9 (10.4)
Male gender, N (%)	692 (74.8%)	131 (61.8%)	120 (43.6%)	69 (51.9%)	105 (58.7%)	105 (46.7%)	1314 (60.3%)
In a relationship, N (%)	99 (10.7%)	30 (14.2%)	89 (32.4%)	35 (26.3%)	21 (11.7%)	41 (18.2%)	324 (14.9%)
Employed, N (%)	62 (6.7%)	31 (14.6%)	43 (15.6%)	16 (12.0%)	22 (12.3%)	19 (8.4%)	200 (9.2%)
Illness duration, mean (SD)	11.0 (8.8)	15.2 (10.1)	14.3 (9.8)	9.9 (9.4)	10.9 (8.7)	12.8 (8.7)	12.0 (9.3)
Comorbid diagnosis, mean (SD)	0.2 (0.4)	0.4 (0.5)	0.3 (0.5)	0.7 (0.5)	0.5 (0.5)	0.4 (0.5)	0.3 (0.5)
GAF, mean (SD)	56.9 (14.7)	59.3 (13.2)	63.5 (12.2)	63.1 (12.9)	62.4 (14.1)	57.9 (12.0)	59.1 (14.0)
CGI, mean (SD)	4.2 (1.1)	4.1 (1.1)	3.7 (1.1)	3.7 (1.1)	3.6 (1.1)	4.1 (1.1)	4.0 (1.1)
**SQoL dimensions, mean (SD)**							
SEL	51.5 (28.3)	49.9 (30.4)	43.0 (28.6)	32.7 (25.6)	37.6 (28.1)	31.7 (25.2)	45.2 (29.1)
ROM	33.1 (29.1)	38.7 (32.7)	35.3 (30.7)	32.3 (30.4)	33.0 (31.7)	31.8 (32.8)	33.7 (30.6)
RES	58.7 (26.5)	61.1 (25.9)	56.3 (27.1)	49.8 (27.4)	52.0 (26.2)	51.4 (27.2)	56.4 (27.0)
PSY	55.4 (26.2)	50.5 (26.2)	51.3 (25.4)	47.6 (25.1)	49.1 (26.2)	46.0 (24.9)	51.5 (26.3)
PHY	48.0 (27.6)	50.0 (29.4)	41.5 (27.0)	34.5 (24.7)	36.7 (26.3)	39.5 (29.0)	44.2 (28.0)
FRI	50.1 (30.2)	53.5 (30.1)	50.6 (29.2)	48.0 (30.3)	44.8 (32.3)	45.8(30.1)	49.4 (30.6)
FAM	68.0 (27.7)	66.0 (28.4)	62.2 (29.8)	59.9 (29.7)	61.5 (29.4)	51.7 (32.0)	63.7 (29.7)
AUT	62.4 (26.3)	62.4 (27.9)	61.7 (25.2)	58.6 (24.9)	55.9 (26.6)	56.2 (25.7)	60.5 (26.4)

*QoL* Quality of life dimensions: *SEL* self-esteem, *ROM* romantic life, *RES* resilience, *PSY* psychological well-being, *PHY* physical well-being, *FRI* relationships with friends, *FAM* family relationships, *AUT* autonomy, *GAF* Global Assessment of Functioning, *CGI* Clinical Global Impression.

### Analysis

#### Network estimation

A network conceptualizes the eight items of the S-QoL as a system of mutually interacting dimensions^18^. Such networks contain nodes (individual items) and edges (associations among individual items). Here, network structures of QoL scores were estimated separately for each of the six mental disorders mentioned above.

One possible way of representing a psychological network is to use the concept of Gaussian graphical model, where nodes represent observed variables, and edges are partial correlation coefficients between two nodes, while controlling for all other nodes in the network^29^. Our network model of QoL dimensions contained a relatively high number of potential edges (36 edges). As is often the case in psychological research, fully saturated parametric regressions aiming to quantify these statistical relationships would quickly break down, because of a limited number of observations given the relatively high number of parameters to be estimated. To palliate for this so-called “curse of multi-dimensionality”, psychological networks make use of the graphical least absolute shrinkage and selection operator procedure (glasso^30,31^). This procedure limits the total sum of absolute parameter values and shrinks partial correlations such that very small edges (likely due to noise) are pushed to zero and thus removed from the network. This encourages the selection of simple, sparse models (i.e. with fewer edges). The amount of shrinkage is defined by a tuning parameter lambda used in the glasso procedure.

Technically, the glasso is run for 100 values of the tuning parameter, logarithmically spaced between its maximal value at which all edges are zero. The graph with the minimal extended Bayesian information criterion (EBIC^32^), which penalizes maximum likelihood estimation by taking into account both the number of edges and the complexity (size) of the model space, is then selected. The strength of the latter penalty depends on the value of a so-called hyperparameter gamma, which we set to 0.5 as recommended in psychological network studies with continuous variables^33^. Again, this procedure aims at selecting the optimal sparse network model.

Gaussian graphical models usually assume a multivariate normal density of the data. Yet, our observed data arises from items on self-report instruments that use rating scales with a small number of response options. Therefore, we used the polychoric correlation technique that aims to estimate the correlation between two hypothesised normally distributed continuous latent variables, from two observed ordinal variables^34^.

Ultimately, the glasso procedure provides a way to visually and statistically assess a network structure, that is, a network where edges are present (not shrunk, i.e. different from 0) vs. absent (shrunk, not significantly different from 0). Network estimation also provides a way to visually assess the local strength of each edge (the strength of the relationship between two nodes), and the global strength of the network (its overall connectivity, i.e. taking into account the strength of all edges).

#### Centrality indices

To gain more insight on the importance of individual QoL dimensions in the networks, we measured so-called centrality indices for each node in each network. Three centrality measures are typically used in network analysis: node strength, closeness and betweenness^35^. Briefly, node strength quantifies how well a node is directly connected to other nodes; closeness quantifies how well a node is indirectly connected to other nodes; and betweenness quantifies how important a node is in the average path between two other nodes (for a more in-depth definition of those centrality measures, please refer to^36^).

Among those three measures, only node strength satisfied our accuracy tests criteria consistently across diagnosis groups (see “Accuracy tests”). Therefore, we chose to only interpret node strength as a centrality index in the current analysis.

#### Tests on within-group edge-weights and centrality indices

For each network, we tested for significant differences between edge-weights (connections between nodes) and centrality indices (of each node) using bootstrapping procedure^19^. To do so, we took the difference between bootstrapped values of one edge-weight or centrality and another edge-weight or centrality, and constructed a bootstrapped confidence interval (CI) around those difference scores. We concluded that two edge-weights or two centrality indices were not significantly different if zero was in the bootstrapped CI^19^.

#### Between-group differences

We assessed between-group differences by testing whether the following four measures were different among the six diagnosis groups: network structure; absolute sum of edge-weights (i.e. overall connectivity); single edge-weight; centrality of each node. We checked for such differences by means of the network comparison test (NCT) developed by van Borkulo et al.^36,37^. This 2-tailed permutation test randomly regroups individuals from each diagnosis group repeatedly (1000 times) and calculates the differences in each of these measures between those samples. The resulting distribution under the null hypothesis that samples are equal is used to test the observed difference of the original samples against a significance level of 0.05, which we corrected to account for multiple comparisons using the Holm procedure.

#### Accuracy tests

We ran the following accuracy analysis to obtain confidence intervals around edge-weights and confirm the stability of our centrality measures, and therefore improve the robustness of our interpretations^19^.

First, we estimated 95% confidence intervals to assess the variability of each edge-weight by means of the non-parametric bootstrap. Second, we also used bootstrapping procedures to determine the stability of each centrality measure. Briefly, with stability we indicate if the order of centrality indices remains the same after re-estimating the network with fewer cases. To quantify the stability of centrality indices using subset bootstraps, we assess the maximum proportion of cases that can be dropped, such that with 95% probability the correlation between original centrality indices and centrality of networks based on subsets is not below 0.7. Under this criteria, we defined stable centrality indices as those for which at least 50% of cases can be removed^19^.

Analyses were conducted with R version 4.0 and packages *qgraph*, *glasso*, *bootnet*, and* NetworkComparisonTest*.

### Ethical standards

The authors assert that all procedures contributing to this work comply with the ethical standards of the relevant national and institutional committees on human experimentation and with the Helsinki Declaration of 1975, as revised in 2008. The REHABase database obtained the authorizations required under French legislation (French National Advisory Committee for the Treatment of Information in Health Research, 16.060bis; French National Computing and Freedom Committee, DR-2017-268). Written informed consent to have their data included in the database was obtained from participants.

## Results

For each disorder investigated in the current study, we first aimed to estimate a network of eight quality-of-life dimensions (nodes) as measured by the Schizophrenia Quality of Life-18 questionnaire (S-QoL18): self-esteem (SEL), romantic life (ROM), resilience (RES), psychological well-being (PSY), physical well-being (PHY), relationships with friends (FRI), relationships with family (FAM), autonomy (AUT). Second, our centrality analysis provided a measure of the importance that each node had in the network, in terms of its relationships with other nodes (using a measure called “node strength”). Third, we ran within-group tests aiming to compare, for each network, edge-weights (measuring the strength of the relationship between two nodes) and nodes’ strength. Fourth, between-group tests aimed at comparing, between diagnosis groups, the structure of the networks, their overall connectivity, local edge-weights, and node strength.

### Network estimation

**Figure 1:**
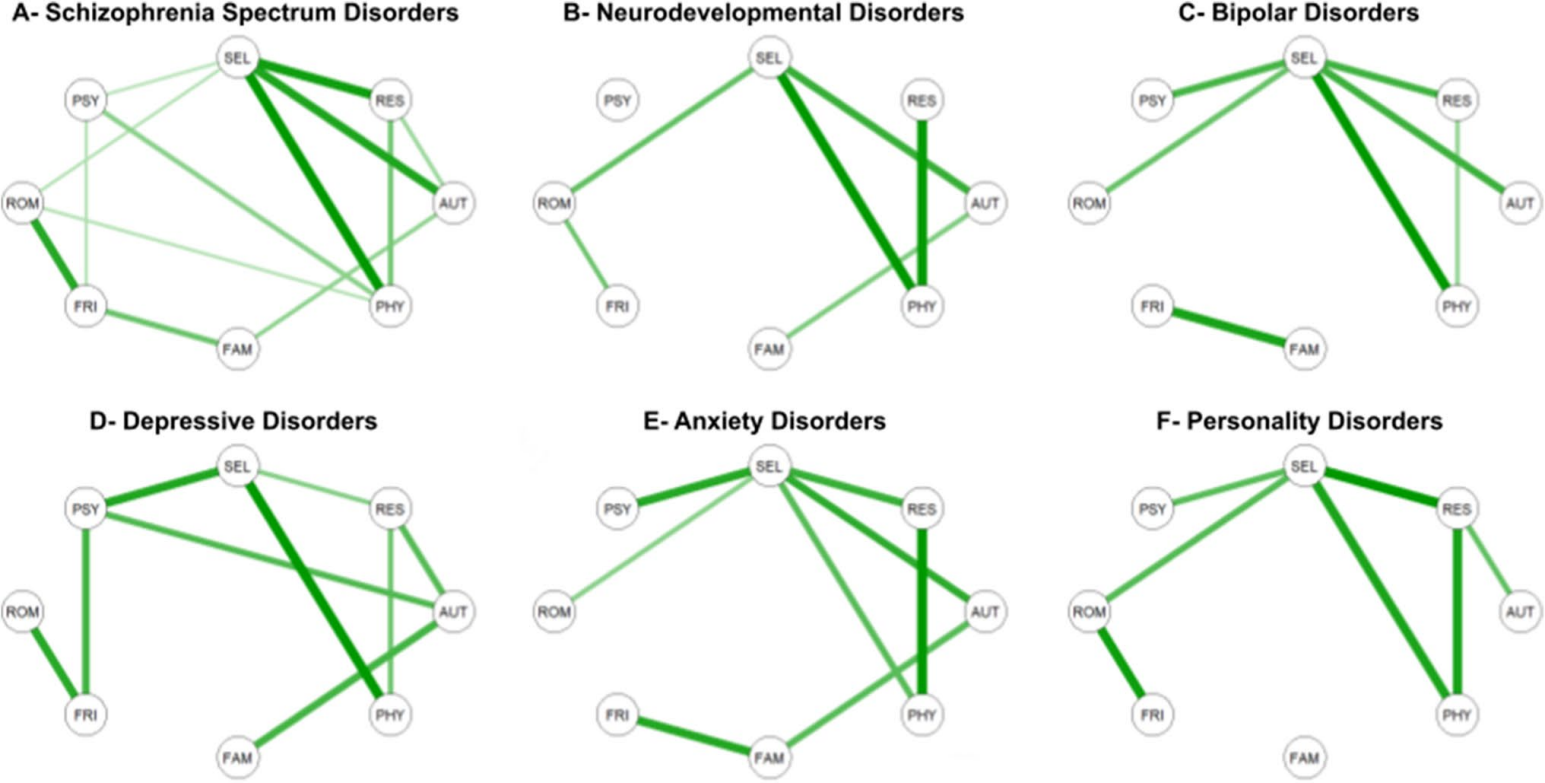
Estimated network structure of quality of life dimensions for the six diagnosis groups. For each diagnosis group (**A**–**F**), the network is a Gaussian graphical model, i.e. a network of partial correlation coefficients, in which glasso regularization is applied. Quality of life dimensions: *SEL* self-esteem, *ROM* romantic life, *RES* resilience, *PSY* psychological well-being, *PHY* physical well-being, *FRI* relationships with friends, *FAM* family relationships, *AUT* autonomy.

In terms of network structure and overall connectivity, psychological networks looked relatively similar across diagnosis groups (Fig. 1; we also provided a visualization of the psychological network where all patients from the REHABase cohort were aggregated in Supplementary Material 1). Visual inference should be done with care, yet most edges that involved self-esteem (SEL), such as SEL–PHY, were consistently represented across diagnosis groups, with the exception of SEL–FRI and SEL–FAM. The latter were consistently pushed to 0 by the LASSO procedure, which was also the case for other edges not involving SEL, e.g. PSY–ROM, or PHY–AUT.

### Centrality analysis

**Figure 2:**
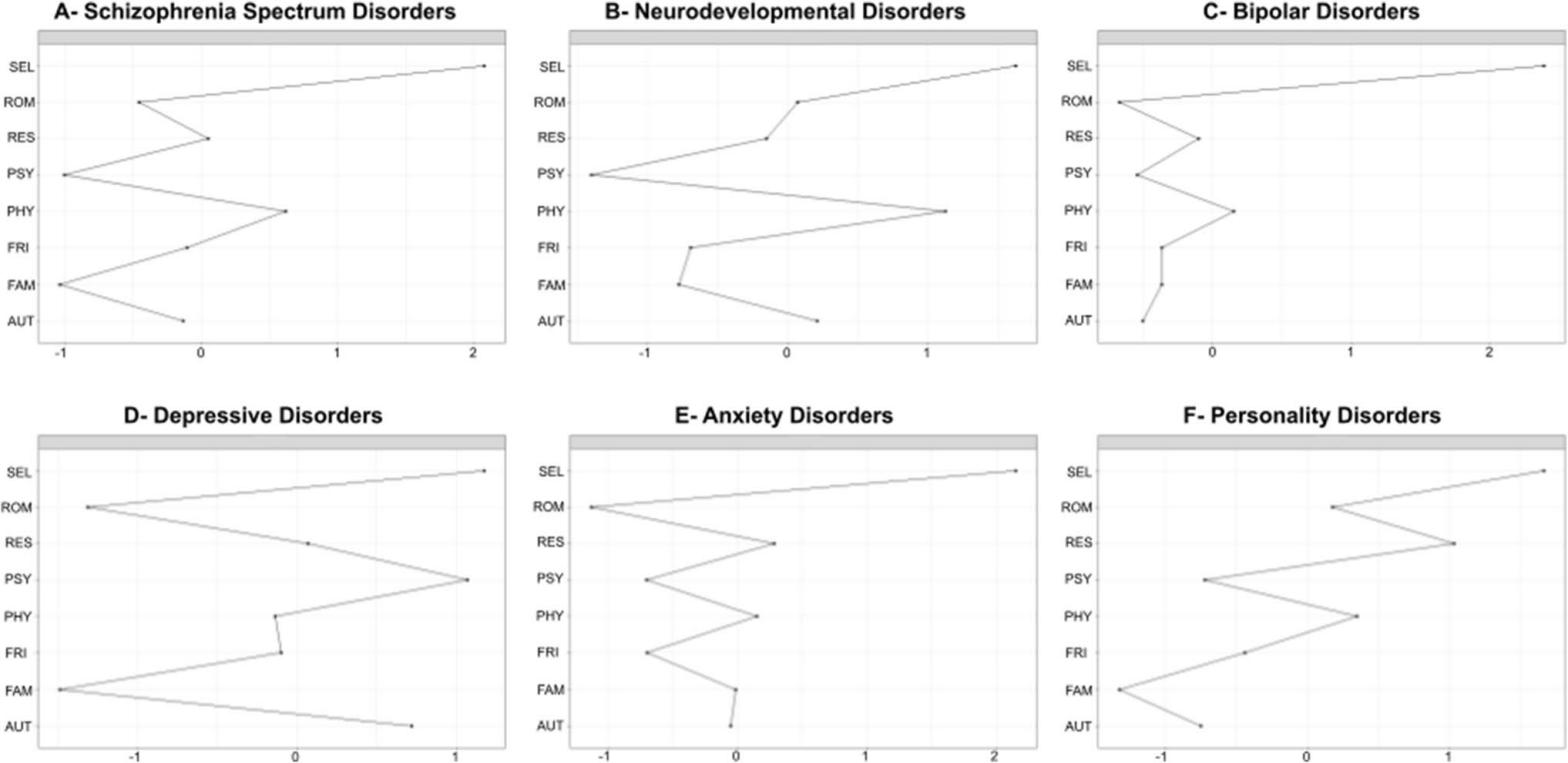
Centrality indices (node strength) of the eight quality of life dimensions obtained for the six diagnosis groups. For each diagnosis group (**A**–**F**), each node strength is shown as a standardized z-score (X axis). Quality of life dimensions (Y axis): *SEL* self-esteem, *ROM* romantic life, *RES* resilience, *PSY* psychological well-being, *PHY* physical well-being, *FRI* relationships with friends, *FAM* family relationships, *AUT* autonomy.

Across diagnosis groups, node strength was the most reliable of the three centrality indices measured in the current study (see point 3. below for an assessment of reliability regarding node strength, closeness and betweenness). Therefore, we chose to only report results on node strength for our centrality analysis. Centrality plots looked relatively similar across diagnosis groups (Fig. 2; we also provided a centrality plot where all patients from the cohort were aggregated in Supplementary Material 1). In particular, for each diagnosis group the self-esteem node (SEL) was consistently stronger than any other nodes. In contrast, PSY, ROM and FAM presented with the smallest node strength in most diagnosis groups (Fig. 2).

### Within-group tests on edge-weights and centrality measures

**Figure 3:**
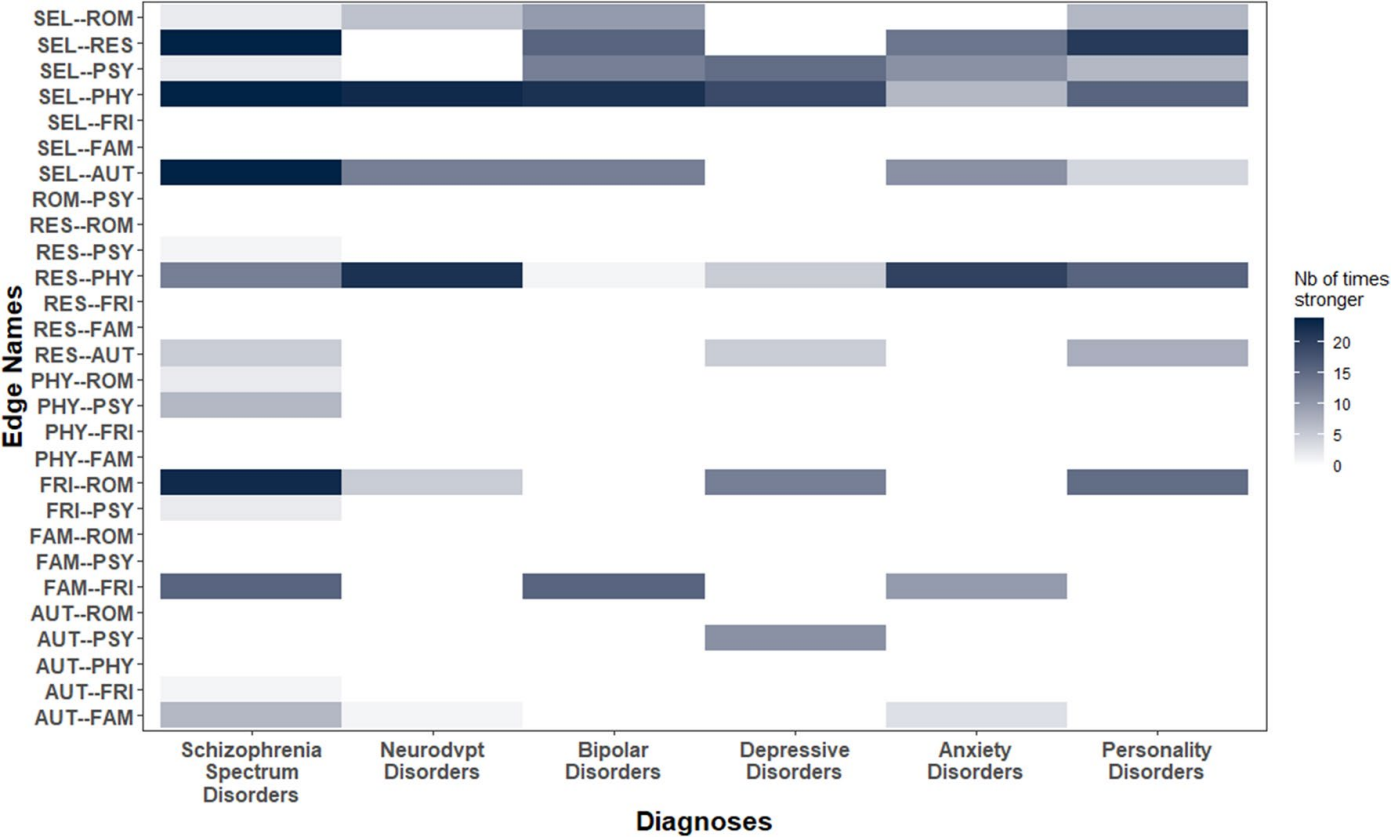
Relative importance of edges connecting quality of life dimensions among the six diagnosis groups. For each diagnosis group (X axis), each tile represents the number of times each edge (Y axis) shows significantly greater weight than other edges. Quality of life dimensions: *SEL* self-esteem, *ROM* romantic life, *RES* resilience, *PSY* psychological well-being, *PHY* physical well-being, *FRI* relationships with friends, *FAM* family relationships, *AUT* autonomy.

For each network, we found that edges involving self-esteem (SEL), including SEL–PHY, SEL–AUT, SEL–PSY, SEL–RES were consistently and significantly stronger than most other edges (Fig. 3; see also Supplementary Material 2 for 95% confidence intervals around each edge-weight; and Supplementary Material 3 for within-groups edge-weights comparison tests). Other relatively strong edge-weights were: RES–PHY, FRI–ROM and FAM–FRI (Fig. 3; Supplementary Materials 2 & 3).

**Figure 4:**
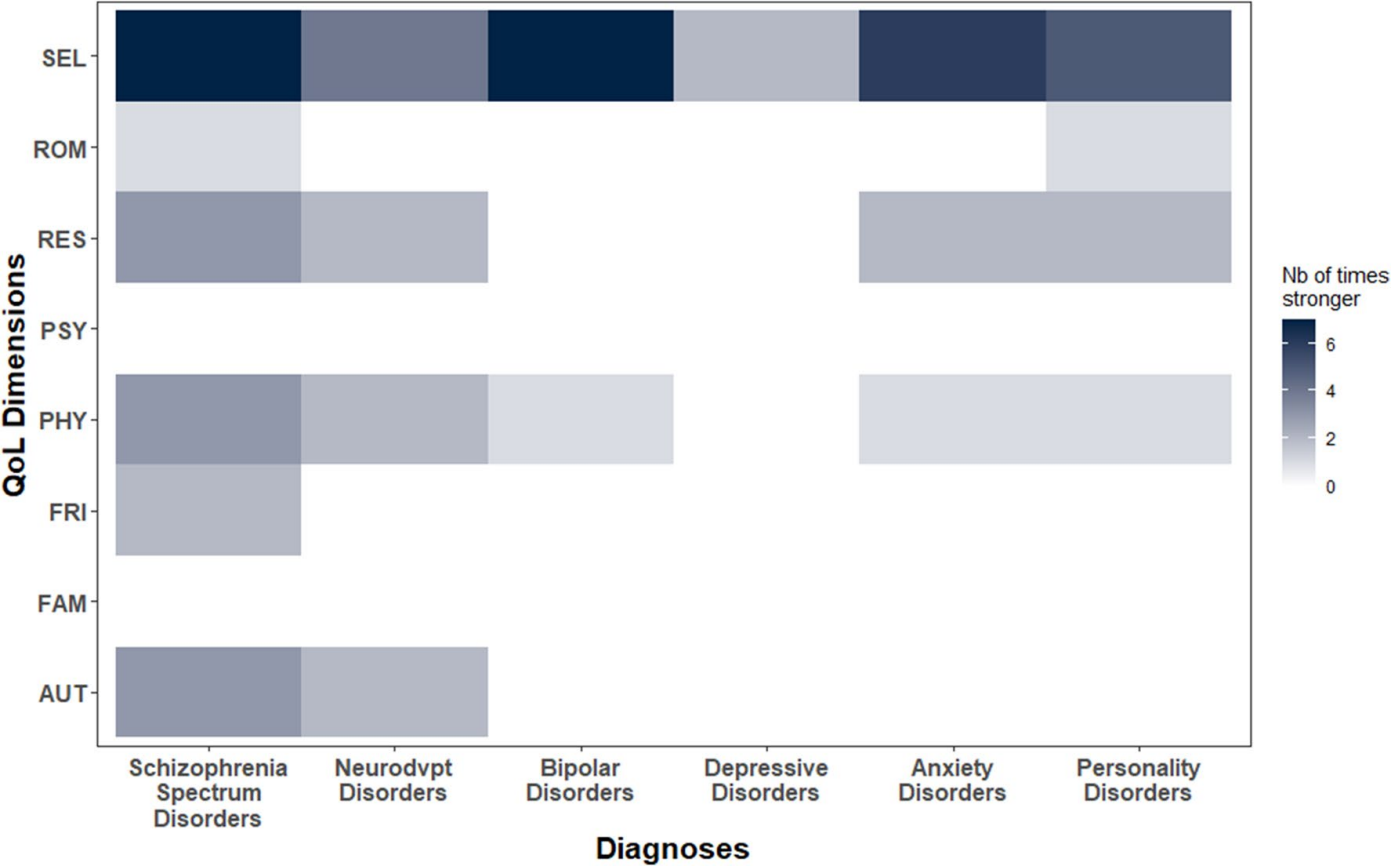
Relative importance of quality of life dimensions (nodes) among the six diagnosis groups. For each diagnosis group (X axis), each tile represents the number of times each node (Y axis) shows significantly greater strength than other nodes. Nodes (Quality of life dimensions; Y axis): *SEL* self-esteem, *ROM* romantic life, *RES* resilience, *PSY* psychological well-being, *PHY* physical well-being, *FRI* relationships with friends, *FAM* family relationships, *AUT* autonomy.

In general, node strength was the most stable of all centrality measures across all diagnosis groups (Supplementary Material 4). Specifically, the maximum proportion of cases that could be dropped such that the correlation between original centrality indices and indices of subset bootstraps is above 0.7, was consistently higher for node strength (mean: 0.46; sd: 0.19; range 0.20–0.75) than for closeness (mean: 0.30; sd: 0.19; range 0–0.52) and betweenness (mean: 0.27; sd: 0.16; range 0.05–0.52). We therefore concluded that node strength was the most reliable of our centrality indices and chose to only interpret its order rather than that of closeness or betweeneness. Taking this criteria into account, self-esteem (SEL) consistently demonstrated greater node strength across diagnosis groups, followed by PHY and RES (Fig. 4; Supplementary Material 5).

### Between-group differences

Significant differences retrieved by our network comparison tests were scarce. In particular, there were no significant between-group differences in terms of overall structure (all p’s > 0.08) and node strength (all p’s > 0.15). Only four tests demonstrated a small level of significance (p’s > 0.03; all other p’s > 0.10). Two of these tests compared between-networks overall connectivity (bipolar vs. schizophrenia spectrum disorders; and bipolar vs. neurodevelopmental disorders). The remaining two tests that were significant compared edge-weights (bipolar vs. depressive disorders for edge AUT–PSY; and personality vs. depressive disorders for edge AUT–FAM) (Supplementary Material 6).

## Discussion

In the current study, we used a French multicentric database of psychiatric patients (REHABase^20^), in conjunction with psychological networks theory^17,18^, to investigate the relationships between quality-of-life dimensions in six relatively common and severe psychiatric disorders. Psychological networks provide an alternative, perhaps more ecological way of representing psychological suffering, where the investigation focuses on a set of interacting dimensions, rather than a specific one (or sum of ones). Psychological networks conceptualize dimensions as nodes that have more or less connectivity with other nodes in the network—such local connectivity is called edge-weight. A crucial step in a psychological network analysis is to run accuracy tests which investigate the reliability of the results^19^. In addition to its innovative design, one strength of the current study is that our accuracy tests all provided strong evidence towards the robustness of our results. Below, we discuss two important findings of our study: (1) networks were comparable across disorders in terms of their overall structure and connectivity, as well as specific edge-weights and node strength; (2) as a quality-of-life dimension, self-esteem was consistently retrieved as the strongest node within each network, and was involved in the most important edges.

### Between-group comparison of quality-of-life networks

First, the overall structure and overall connectivity of our psychological networks were not statistically different among diagnosis groups. While diagnoses differ based on their symptom profiles, previous studies had questioned the validity of diagnosis differences in terms of psychopathology measures, socio-demographic predictors, biochemical and genetic disturbances^4,34,38–44^. Accordingly, our findings suggest that quality-of-life dimensions and their inter-connections are relatively similar across psychiatric patients, irrespective of their diagnosis groups. Such commonalities among networks also suggest that quality of life is not the sole product of symptom features, as a typical model of psychiatric disorders being the result of unique underlying entities would posit^17,18^. From this perspective, our findings add to the growing body of studies that aim to decenter psychiatric disorders from symptoms and symptom profiles^2,20,45–48^. We hope that they contribute towards lowering stigma, increased use of psychosocial rehabilitation treatments and social inclusion of those with psychological suffering.

### Self-esteem as the strongest quality-of-life dimension across all diagnosis groups

A second consistent finding of our study is that, across all diagnosis groups, the single strongest node in each quality-of-life network was that of self-esteem. Quality-of-life dimensions that were the most related to self-esteem were physical and psychological well-being, autonomy, and resilience. As the degree to which an individual has a favourable attitude towards the self (a sense of self-worth), self-esteem is core to one’s own actions, emotional and physical fulfilment, coping with daily life, and to start and maintain satisfying relationships. Moreover, in a world where norms place a strong emphasis on the individual, his/her level of joy and self-image^49^, self-esteem is highly solicited and therefore easily perturbed. Our results suggest that this heightened vulnerability of self-esteem inherent to our modern way of life can be framed as a core risk factor for the deterioration of other quality-of-life dimensions, such as psychological and physical well-being, resilience and autonomy, and the deterioration of quality of life in general—a bit like in a house of cards^50,51^.

Taken as a broad concept related to negative self-evaluation, low self-esteem is core to most mental disorders, either as a formal diagnostic criterion (e.g. in depressive disorders), or as an associated feature (e.g. in schizophrenia)^27,52^. Low self-esteem is also considered a transdiagnostic risk and maintenance factor for mental disorders^53–55^. The fact that we did not observe any significant difference in network structure between diagnosis groups does not mean, however, that self-esteem has no discriminatory power among mental disorders. For instance, previous studies have shown that low self-esteem was more pronounced in individuals with major depression and less so in patients with bipolar disorder in the manic phase^52^. Discrepancies with our findings could stem from the fact that patients from the REHABase cohort may be more clinically stable than those who participated in other studies (e.g. stable enough to attend rehab programmes). Other differences with previous research might result from the specificities of our analysis, which investigates the links between self-esteem and other quality-of-life dimensions, rather than self-esteem merely as an outcome. Finally, our analysis did not investigate the mechanisms by which low self-esteem is core to the quality of life of those with mental problems, which may be very different among disorders. Future studies should explore these mechanisms, which may span a broad spectrum of issues, from low income^56^, to internal or external stigmatization^57^, and even high intellectual standing^58^.

Our results could also have interesting therapeutic implications. Even though most treatment guidelines focus primarily on psychiatric disorders rather than on underlying processes or quality-of-life dimensions, many interventions have been developed to specifically target low self-esteem. Among them, it is worth mentioning cognitive-behavioural approaches which aim to identify, challenge and modify dysfunctional automatic thoughts and underlying schemas^59^, and Competitive Memory Training (COMET), which aims at enhancing the retrievability of a patient’s positive self-knowledge^60^. As a central quality-of-life dimension, self-esteem could be considered a fundamental treatment target in psychosocial rehabilitation programmes, in order to improve quality of life in a cost-effective way^61^. Future studies should confirm this hypothesis and test whether psychological work directly targeting self-esteem vs. other dimensions (either other strong nodes, such as resilience and physical well-being, or weaker nodes such as psychological well-being), improves quality of life, functioning and clinical symptoms.

### Limitations

Our study presents several limitations. First, we were only interested in psychological networks of quality-of-life dimensions. Yet, a more ecological representation would probably include relationships with other types of issues, such as clinical symptoms, but also potentially genetic features, past symptomatic history, socio-demographic characteristics, and so on. Clearly, knowledge is lacking on how to best model a bio-psycho-social network of multi-level features among psychiatric disorders.

Second, while the SQoL-18 is a very practical tool to measure quality of life in patients with mental disorders, we acknowledge that its construct and content validity may be limited. This is probably inevitable when one wishes to collect data on such a complex and subjective concept.

Third, to represent our psychological networks, we aggregated individuals with similar diagnosis profiles. Potentially, this could represent a case for the so-called “ecological fallacy”, where characteristics at the group level would bias an individual analysis. As discussed above, our psychological network analysis would benefit from an investigation across various socio-demographic strata. That said, and taking a health services research perspective, our results should be interpreted just as we aimed to design our study: as (broadly) ecologically relevant.

Fourth, as gaussian graphical models, our psychological networks made no assumptions about the direction of connectivity between nodes. Gathering longitudinal information would have been interesting to help us make causal inferences, and further, test the effects of specific treatment programmes. This could be the subject of further studies but would require a different modelling strategy (e.g. with a vector autoregression model to specifically take into account temporality^62^).

Fifth, we acknowledge that in our study, valid statistical inference is challenged by a set of potential issues^19^. Our between-group network comparison tests used permutation tests and took multiple comparisons into account but as currently proposed, could not account for the six diagnosis groups in the same statistical model. Conversely, our within-group tests on edge-weights and centrality indices are not corrected for multiple comparisons, as these bootstrapped-based tests are functions of complicated estimation methods (involving glasso regularization of partial correlation networks). Finally, in simulation studies centrality tests have demonstrated a lower level of power in rejecting the null hypothesis when two centralities differ from one-another^19^. Clearly, these caveats need to be taken into account as our analysis may have suffered from a slight lack of sensitivity and specificity.

## Conclusion

Despite these limitations, which are balanced by a relatively large number of observations and the reliability of our accuracy tests, our study shows that networks of quality-of-life dimensions are in general not significantly different across serious mental illnesses. In addition, our analysis shows that within each group, stronger connections are consistently those that involve self-esteem as a core quality-of-life dimension.

Our study also exemplifies how system thinking techniques can be used to explore dysfunctional psychological dimensions that interact with one-another. Interestingly, these techniques may be more representative of psychological suffering than more analytical tools typically applied in mental health^63,64^. We hope that future studies will continue to use systems science to better describe inter-relations between various features of a patient’s suffering, and test the effectiveness of treatment programmes in an ecological, practical and realistic way.

## Supplementary Information

Supplementary Information 1.

Supplementary Information 2.

Supplementary Information 3.

Supplementary Information 4.

Supplementary Information 5.

Supplementary Information 6.

## Data Availability

Anonymized data generated during and/or analysed during the current study are available upon request to the corresponding author.
