# Supplementary material for: The central role of self-esteem in the quality of life of patients with mental disorders
Source: Sci Rep. 2022 May 12;12:7852. doi: 10.1038/s41598-022-11655-1 (PMC9098638; doi:10.1038/s41598-022-11655-1)
Supplement: Supplementary file 1 — Supplementary Information 1. [file 41598_2022_11655_MOESM1_ESM.pdf]

Supplementary Materials 1. Structure of Quality-of-Life Network (A) and Centrality indices (B) for all patients on the REHABase cohort (N=2180)

A- Estimated network structure of eight quality-of-life dimensions

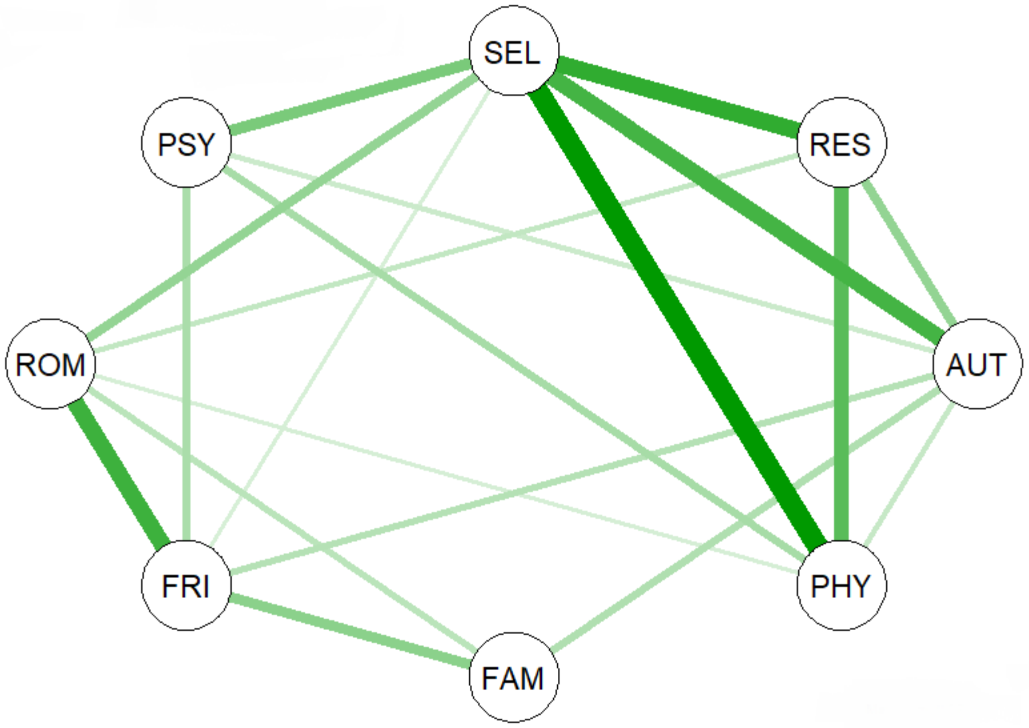

The network is a Gaussian graphical model, i.e. a network of partial correlation coefficients, in which glasso regularization is applied.
Nodes (Quality-of-life dimensions): self-esteem (SEL), romantic life (ROM), resilience (RES), psychological well-being (PSY), physical well-being (PHY), relationships with friends (FRI), family relationships (FAM), autonomy (AUT).

B- Centrality indices (Node strength) of the eight quality-of-life dimensions

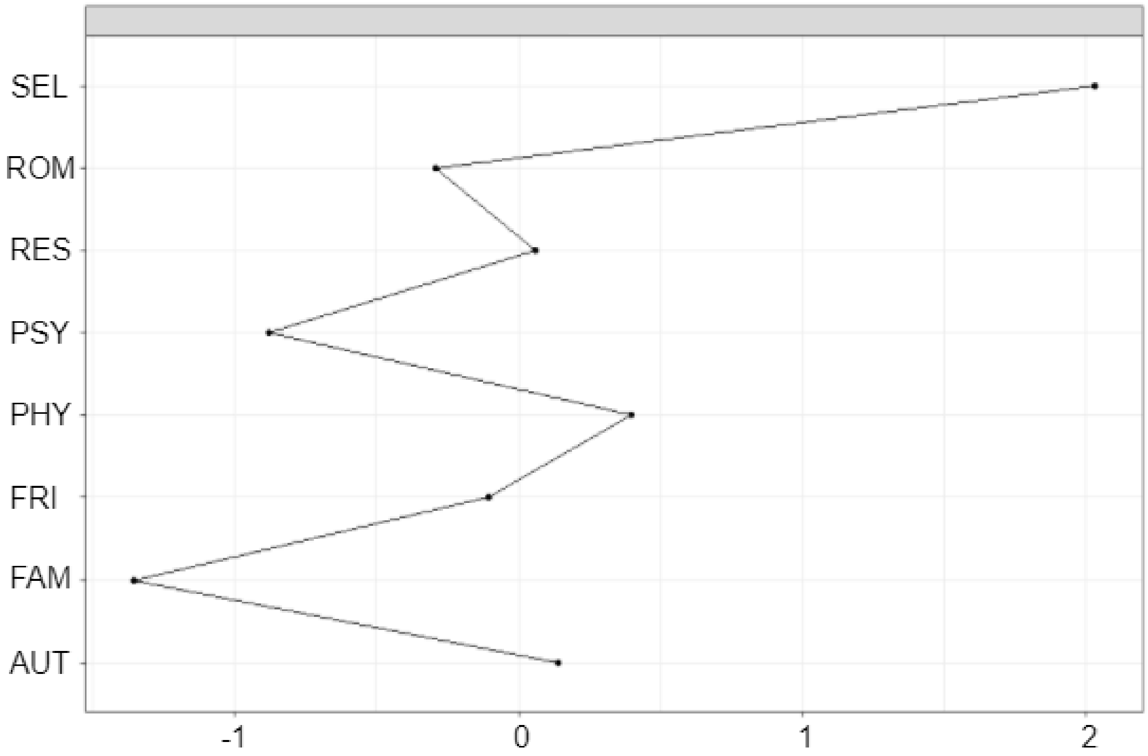

Each node's centrality index is shown as a standardized z-score (X axis; node strength). Nodes (Quality-of-life dimensions; Y axis): self-esteem (SEL), romantic life (ROM), resilience (RES), psychological well-being (PSY), physical well-being (PHY), relationships with friends (FRI), family relationships (FAM), autonomy (AUT).
