# Supplementary material for: The central role of self-esteem in the quality of life of patients with mental disorders
Source: Sci Rep. 2022 May 12;12:7852. doi: 10.1038/s41598-022-11655-1 (PMC9098638; doi:10.1038/s41598-022-11655-1)
Supplement: Supplementary file 2 — Supplementary Information 2. [file 41598_2022_11655_MOESM2_ESM.pdf]

Supplementary Materials 2. Bootstrapped confidence intervals of estimated edge-weights for each estimated network

A- Schizophrenia Spectrum Disorders (N=929)

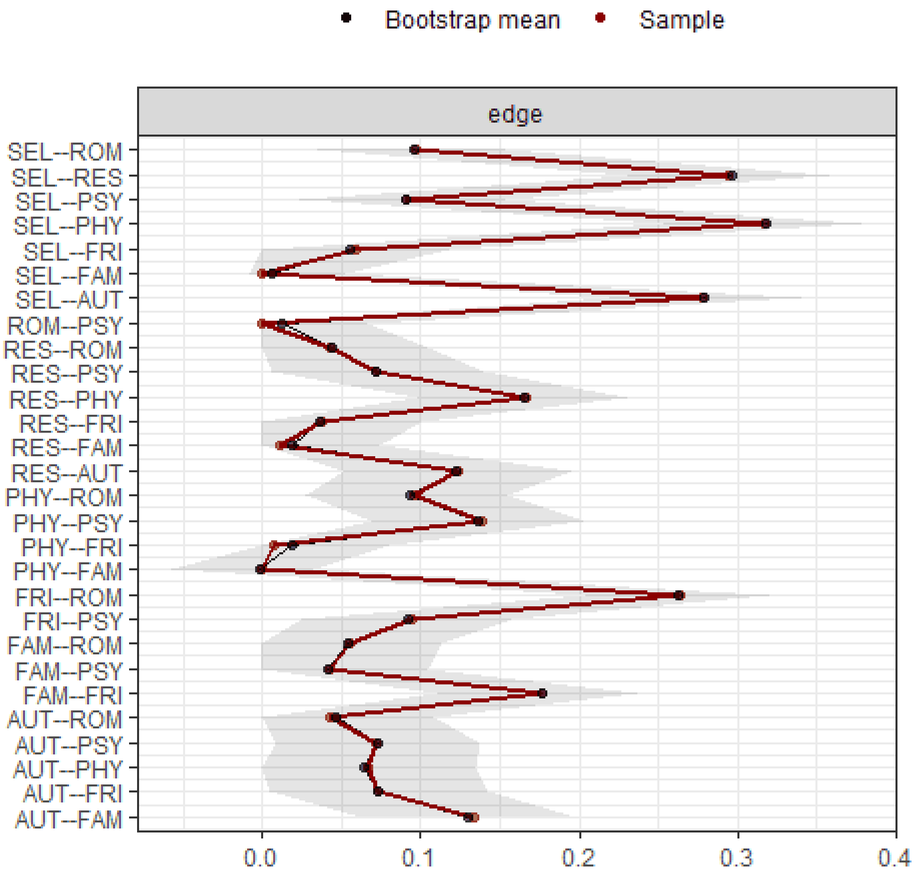

The red line indicates the sample values and the grey area the bootstrapped CIs. Each horizontal line represents one edge of the network, ordered by names.
Quality-of-life dimensions: self-esteem (SEL), romantic life (ROM), resilience (RES), psychological well-being (PSY), physical well-being (PHY), relationships with friends (FRI), family relationships (FAM), autonomy (AUT).

B- Neurodevelopmental Disorders (N=216)

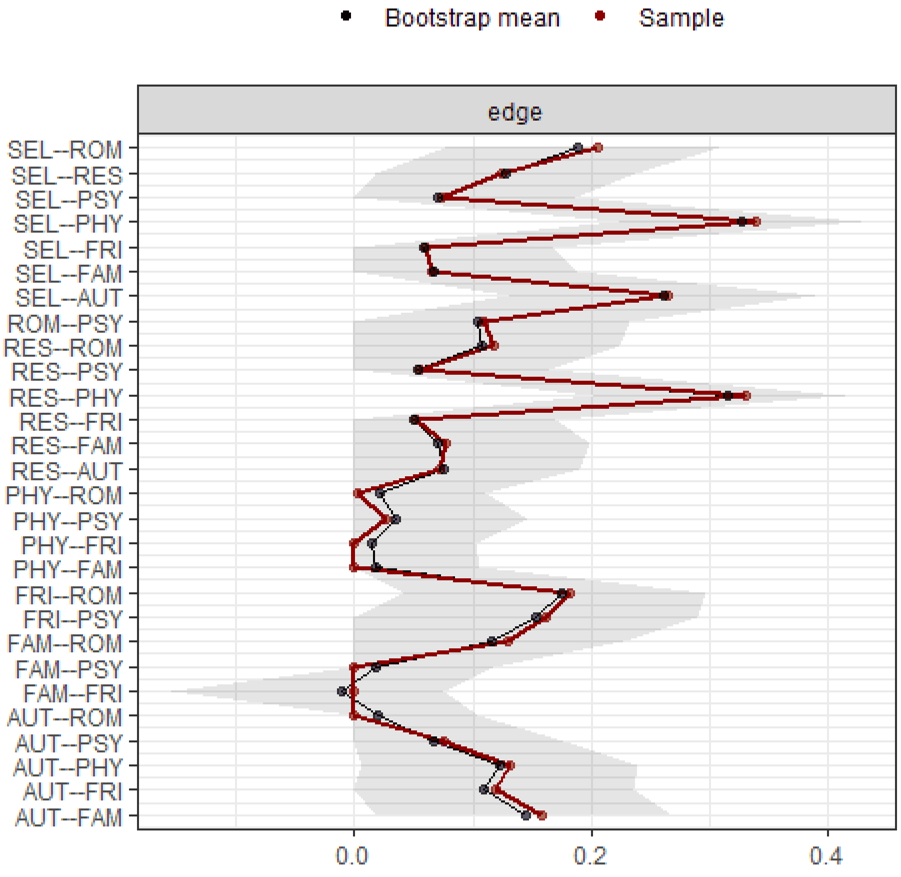

The red line indicates the sample values and the gray area the bootstrapped CIs. Each horizontal line represents one edge of the network, ordered by names (alphabetical order). Quality-of-life dimensions: self-esteem (SEL), romantic life (ROM), resilience (RES), psychological well-being (PSY), physical well-being (PHY), relationships with friends (FRI), family relationships (FAM), autonomy (AUT).

C- Bipolar Disorders (N=275)

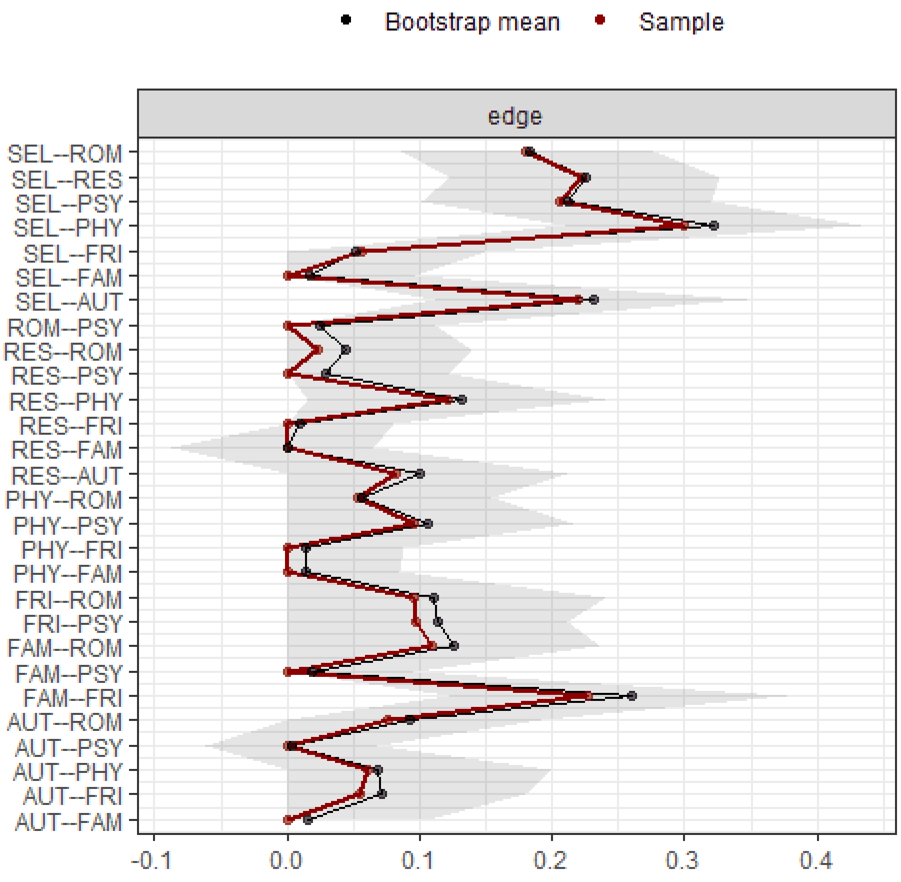

The red line indicates the sample values and the grey area the bootstrapped CIs. Each horizontal line represents one edge of the network, ordered by names (alphabetical order). Quality-of-life dimensions: self-esteem (SEL), romantic life (ROM), resilience (RES), psychological well-being (PSY), physical well-being (PHY), relationships with friends (FRI), family relationships (FAM), autonomy (AUT).

D- Depressive Disorders (N=133)

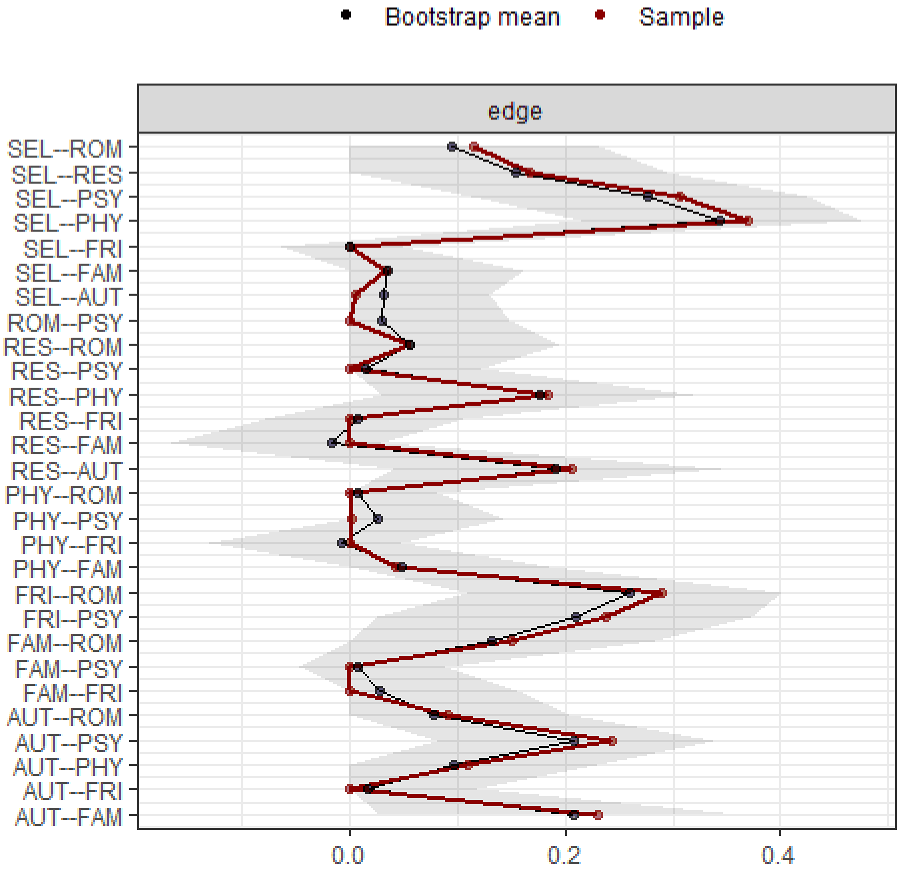

The red line indicates the sample values and the grey area the bootstrapped CIs. Each horizontal line represents one edge of the network, ordered by names (alphabetical order). Quality-of-life dimensions: self-esteem (SEL), romantic life (ROM), resilience (RES), psychological well-being (PSY), physical well-being (PHY), relationships with friends (FRI), family relationships (FAM), autonomy (AUT).

E- Anxiety Disorders (N=179)

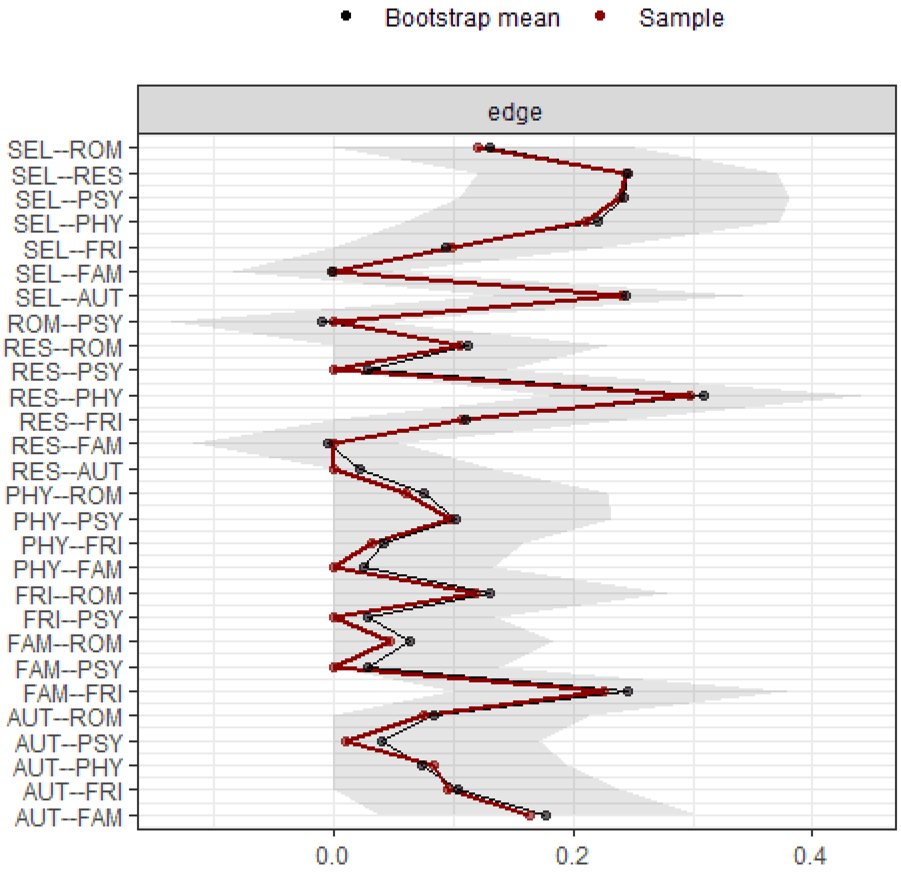

The red line indicates the sample values and the grey area the bootstrapped CIs. Each horizontal line represents one edge of the network, ordered by names (alphabetical order). Quality-of-life dimensions: self-esteem (SEL), romantic life (ROM), resilience (RES), psychological well-being (PSY), physical well-being (PHY), relationships with friends (FRI), family relationships (FAM), autonomy (AUT).

F- Personality Disorders (N=230)

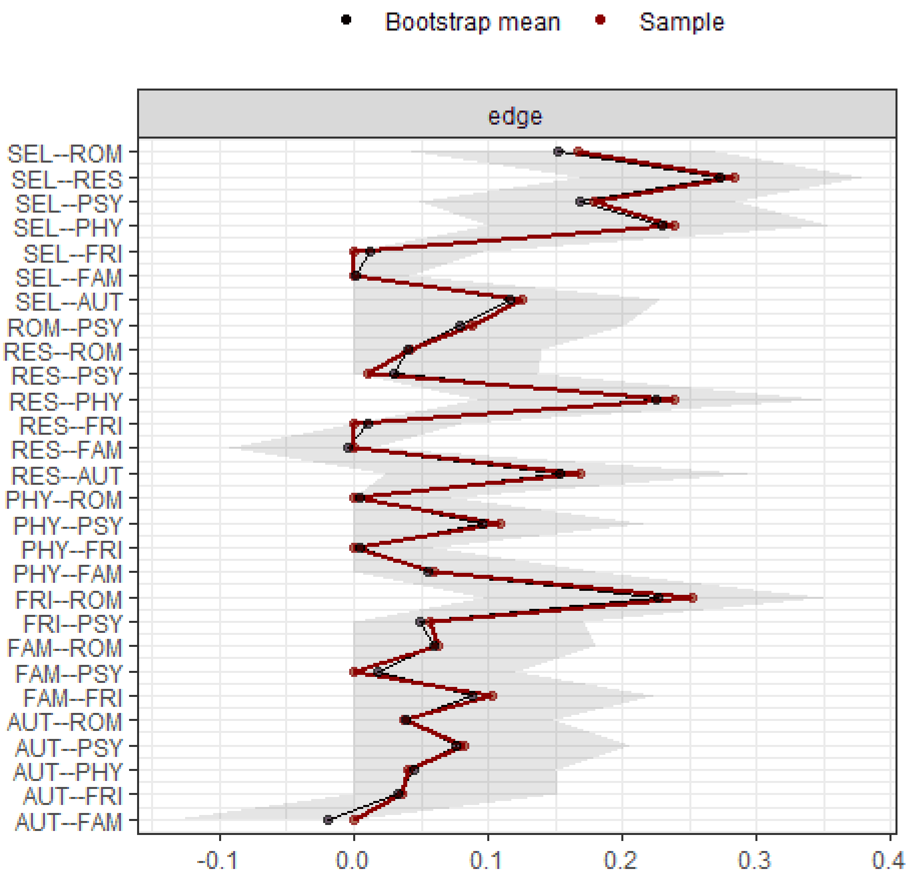

The red line indicates the sample values and the grey area the bootstrapped CIs. Each horizontal line represents one edge of the network, ordered by names (alphabetical order). Quality-of-life dimensions: self-esteem (SEL), romantic life (ROM), resilience (RES), psychological well-being (PSY), physical well-being (PHY), relationships with friends (FRI), family relationships (FAM), autonomy (AUT).
