# Supplementary material for: The central role of self-esteem in the quality of life of patients with mental disorders
Source: Sci Rep. 2022 May 12;12:7852. doi: 10.1038/s41598-022-11655-1 (PMC9098638; doi:10.1038/s41598-022-11655-1)
Supplement: Supplementary file 3 — Supplementary Information 3. [file 41598_2022_11655_MOESM3_ESM.pdf]

Supplementary Materials 3. Bootstrapped difference tests (α = 0.05) between edge-weights that were non-zero in the estimated network

A- Schizophrenia Spectrum Disorders (N=929)

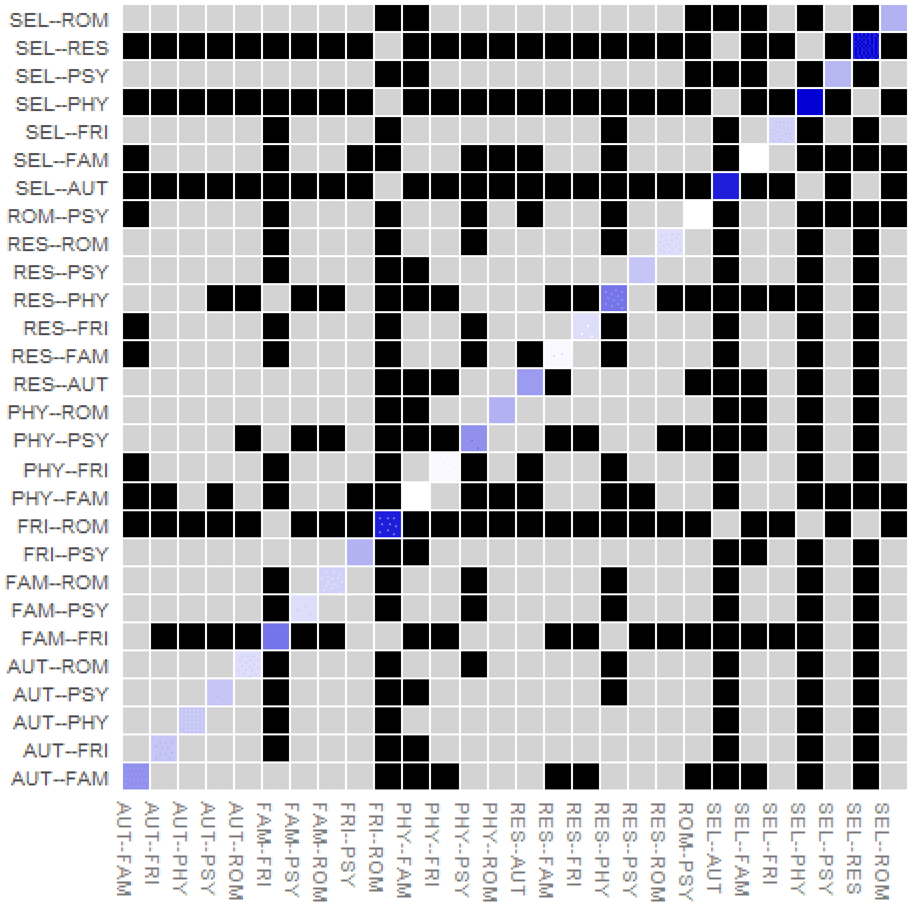

Grey boxes indicate edges that do not differ significantly from one another and black boxes represent edges that do differ significantly from one another. The intensity of the color in the central, diagonal boxes corresponds to the strength of the edge. No correction for multiple testing was applied.
Quality-of-life dimensions: self-esteem (SEL), romantic life (ROM), resilience (RES), psychological well-being (PSY), physical well-being (PHY), relationships with friends (FRI), family relationships (FAM), autonomy (AUT).

B- Neurodevelopmental Disorders (N=216)

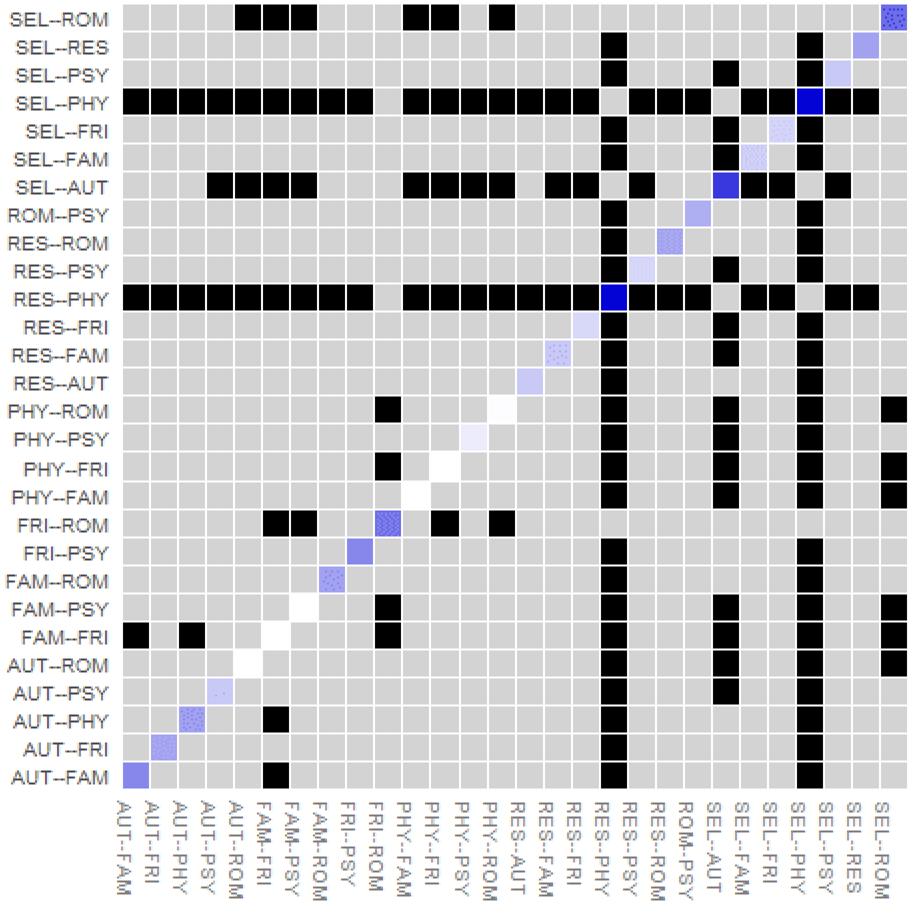

Grey boxes indicate edges that do not differ significantly from one another and black boxes represent nodes or edges that do differ significantly from one another. The intensity of the blue color in central boxes corresponds to the strength of the edge. No correction for multiple testing was applied.
Quality-of-life dimensions: self-esteem (SEL), romantic life (ROM), resilience (RES), psychological well-being (PSY), physical well-being (PHY), relationships with friends (FRI), family relationships (FAM), autonomy (AUT).

C- Bipolar disorders (N=275)

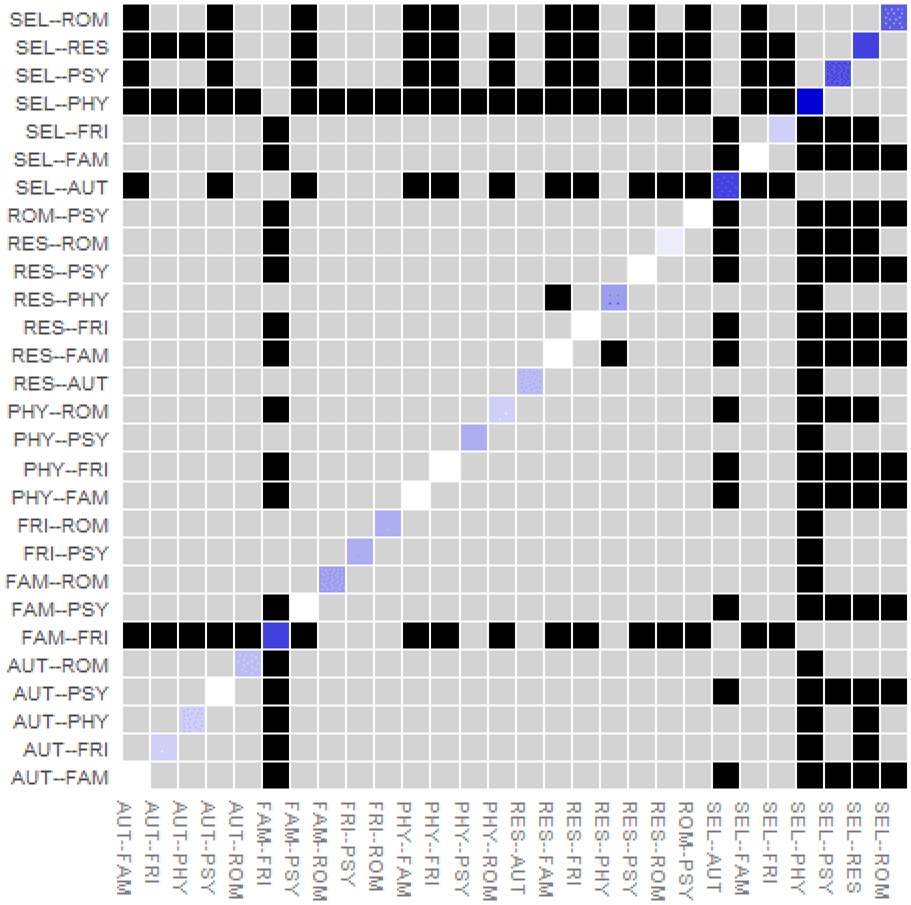

Grey boxes indicate edges that do not differ significantly from one another and black boxes represent edges that do differ significantly from one another. The intensity of the color in the central, diagonal boxes corresponds to the strength of the edge. No correction for multiple testing was applied.
Quality-of-life dimensions: self-esteem (SEL), romantic life (ROM), resilience (RES), psychological well-being (PSY), physical well-being (PHY), relationships with friends (FRI), family relationships (FAM), autonomy (AUT).

D- Depressive disorders (N=133)

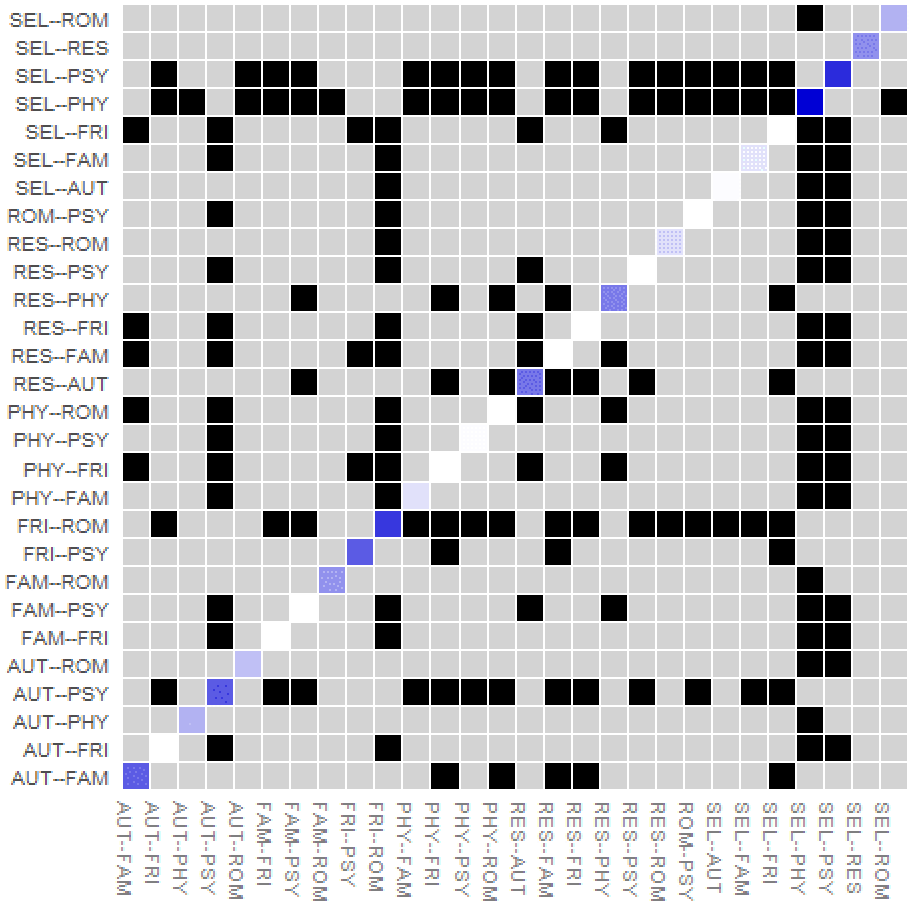

Grey boxes indicate edges that do not differ significantly from one another and black boxes represent edges that do differ significantly from one another. The intensity of the color in the central, diagonal boxes corresponds to the strength of the edge. No correction for multiple testing was applied.

Quality-of-life dimensions: self-esteem (SEL), romantic life (ROM), resilience (RES), psychological well-being (PSY), physical well-being (PHY), relationships with friends (FRI), family relationships (FAM), autonomy (AUT).

E- Anxiety Disorders (N=179)

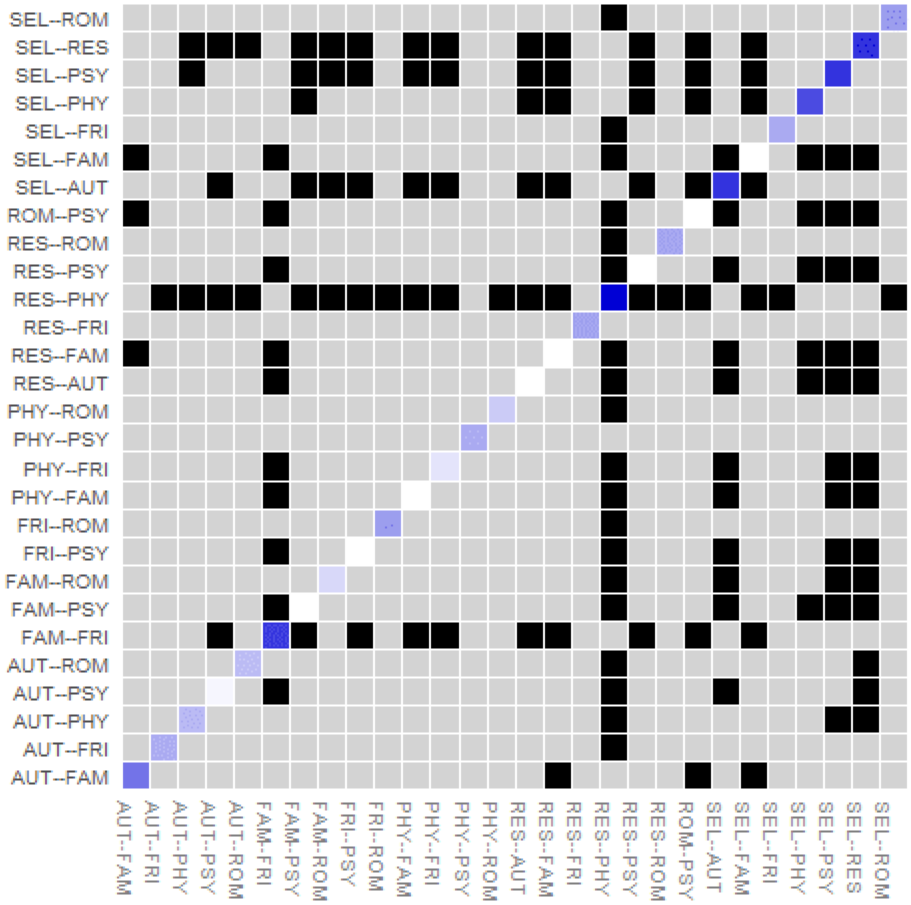

Grey boxes indicate edges that do not differ significantly from one another and black boxes represent edges that do differ significantly from one another. The intensity of the color in the central, diagonal boxes corresponds to the strength of the edge. No correction for multiple testing was applied.

Quality-of-life dimensions: self-esteem (SEL), romantic life (ROM), resilience (RES), psychological well-being (PSY), physical well-being (PHY), relationships with friends (FRI), family relationships (FAM), autonomy (AUT).

F- Personality Disorders (N=230)

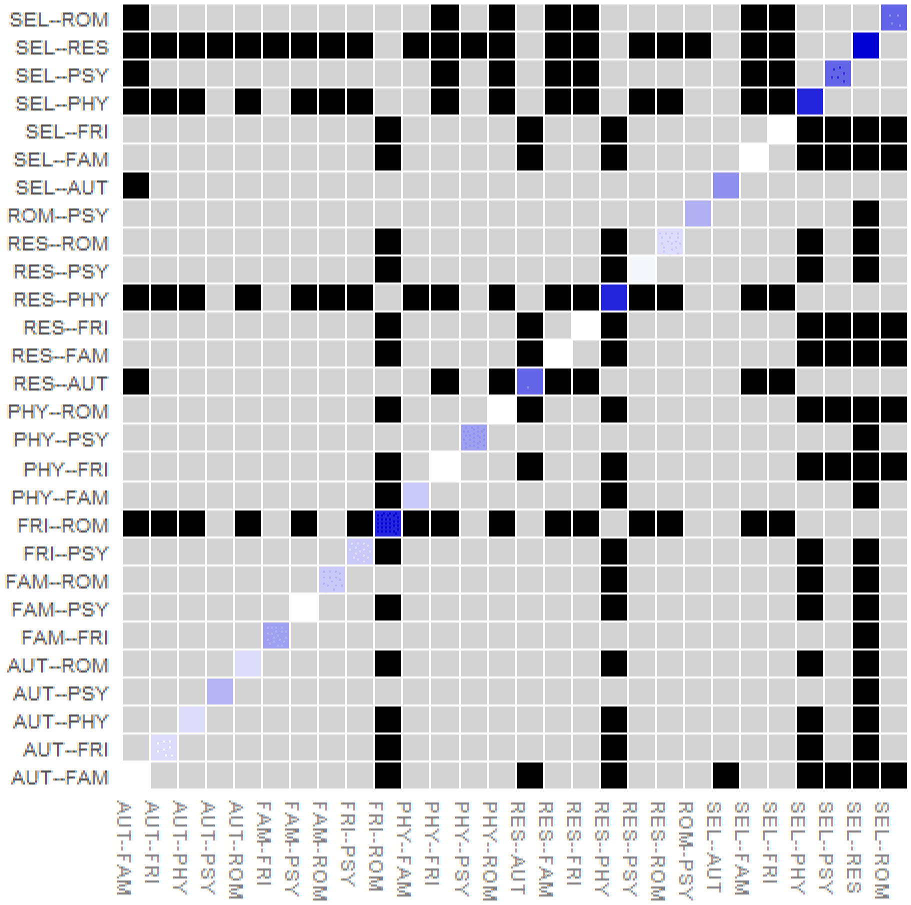

Grey boxes indicate edges that do not differ significantly from one another and black boxes represent edges that do differ significantly from one another. The intensity of the color in the central, diagonal boxes corresponds to the strength of the edge. No correction for multiple testing was applied.
Quality-of-life dimensions: self-esteem (SEL), romantic life (ROM), resilience (RES), psychological well-being (PSY), physical well-being (PHY), relationships with friends (FRI), family relationships (FAM), autonomy (AUT).
