# Supplementary material for: The central role of self-esteem in the quality of life of patients with mental disorders
Source: Sci Rep. 2022 May 12;12:7852. doi: 10.1038/s41598-022-11655-1 (PMC9098638; doi:10.1038/s41598-022-11655-1)
Supplement: Supplementary file 4 — Supplementary Information 4. [file 41598_2022_11655_MOESM4_ESM.pdf]

Supplementary Materials 4. Correlation of centrality indices under different levels of subsetting

A- Schizophrenia Spectrum Disorders (N=929)

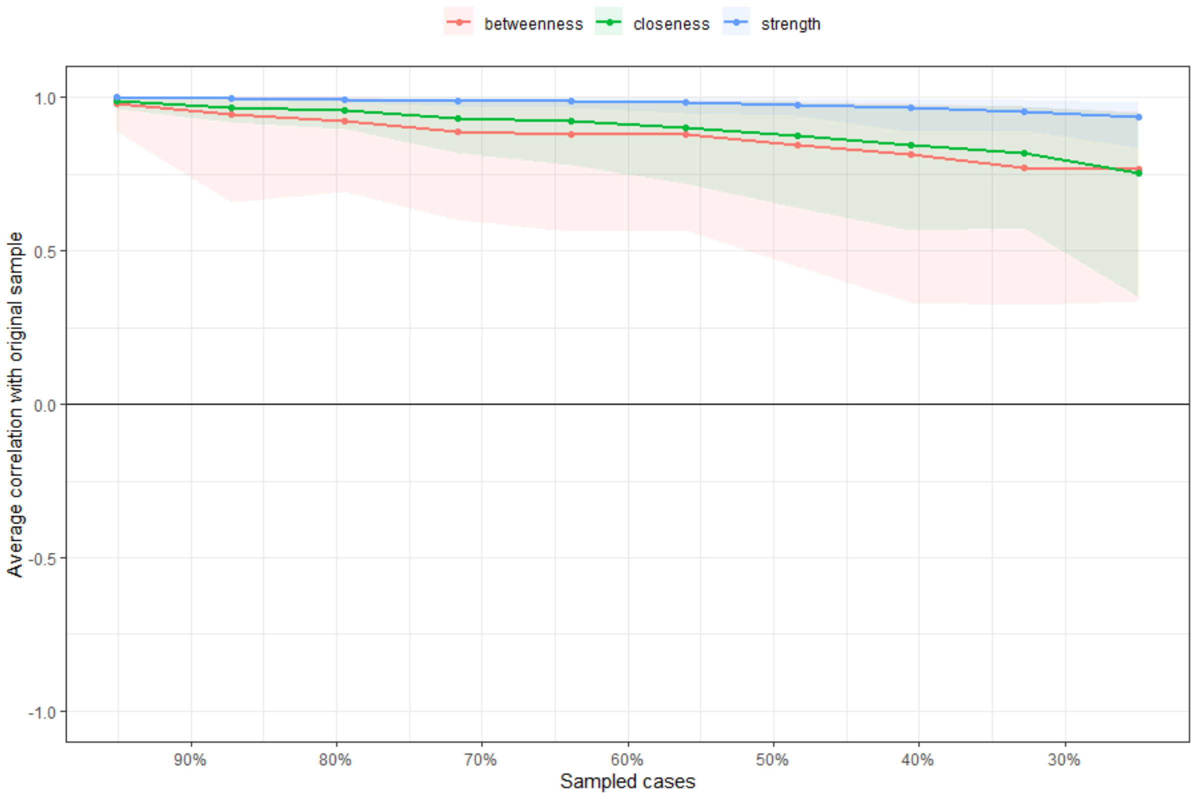

Average correlations between centrality indices of networks sampled with persons dropped and from the original sample. Lines indicate the means and areas indicate the range from the 2.5th quantile to the 97.5th quantile.

B- Neurodevelopmental Disorders (N=216)

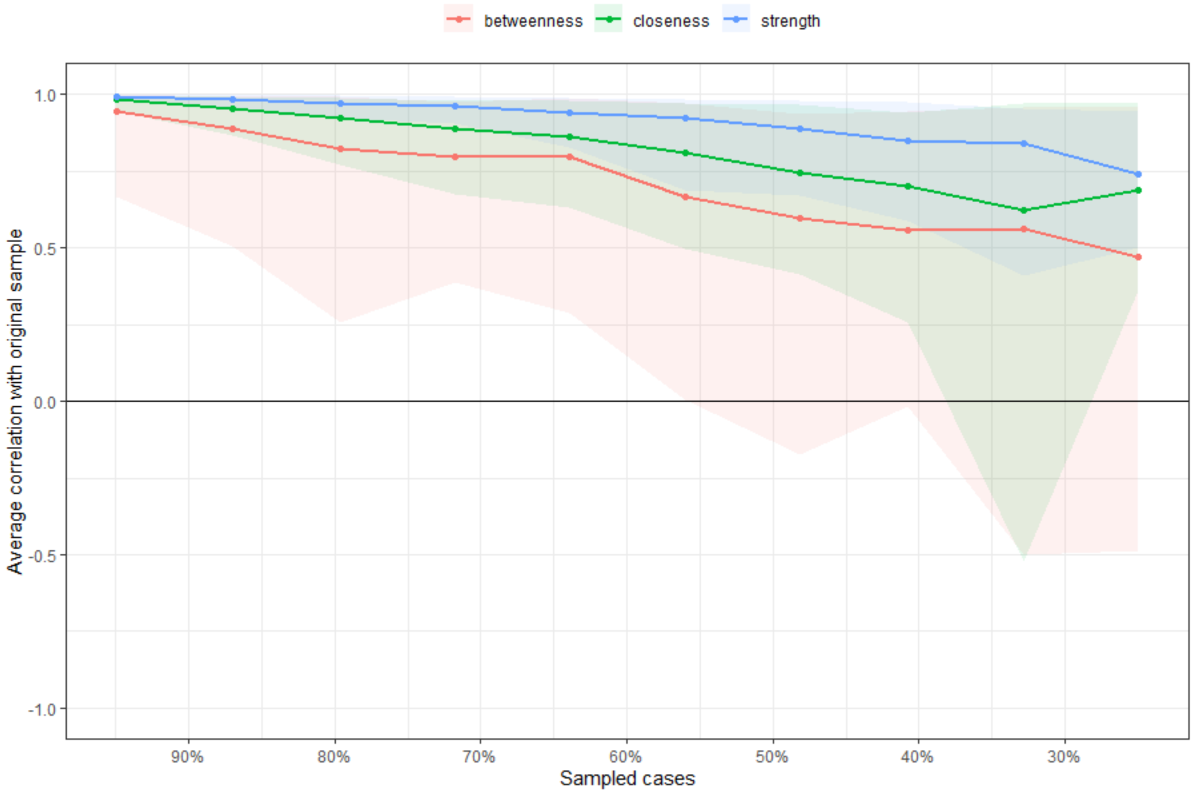

Average correlations between centrality indices of networks sampled with persons dropped and from the original sample. Lines indicate the means and areas indicate the range from the 2.5th quantile to the 97.5th quantile.

C. Bipolar Disorders (N=275)

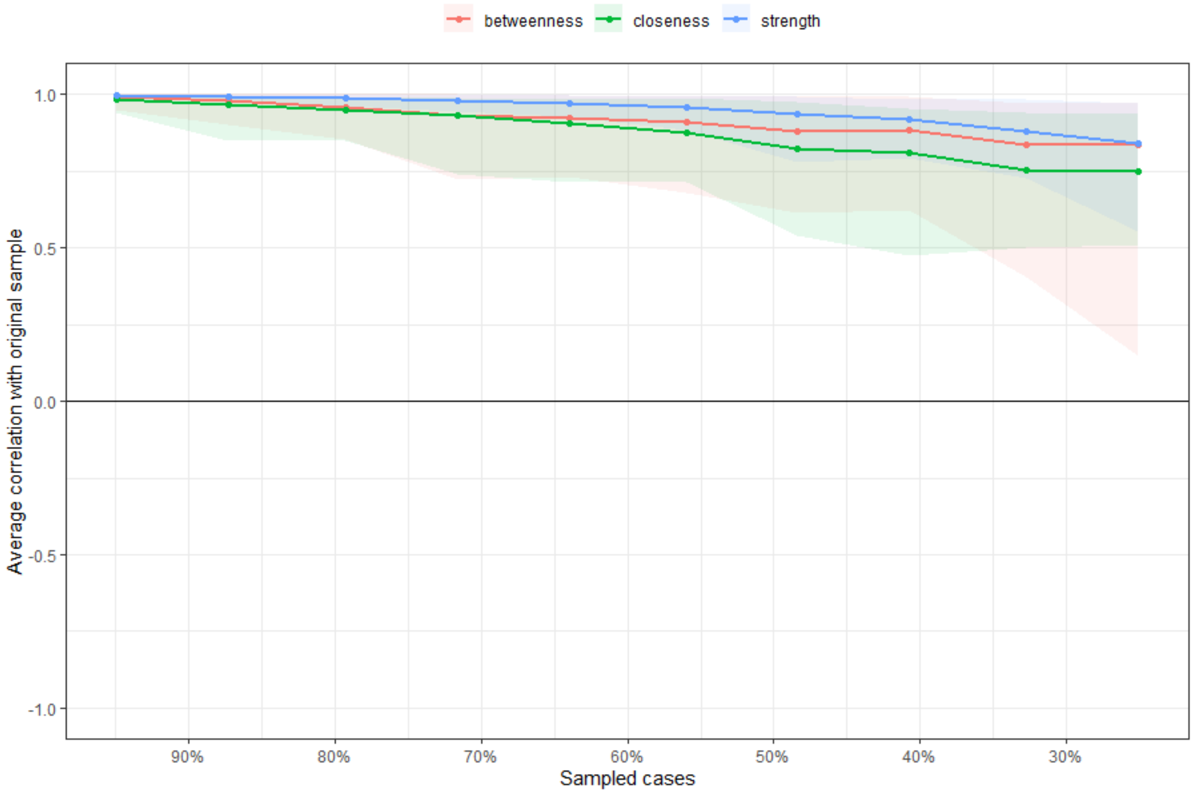

Average correlations between centrality indices of networks sampled with persons dropped and from the original sample. Lines indicate the means and areas indicate the range from the 2.5th quantile to the 97.5th quantile.

D- Depressive Disorders (N=133)

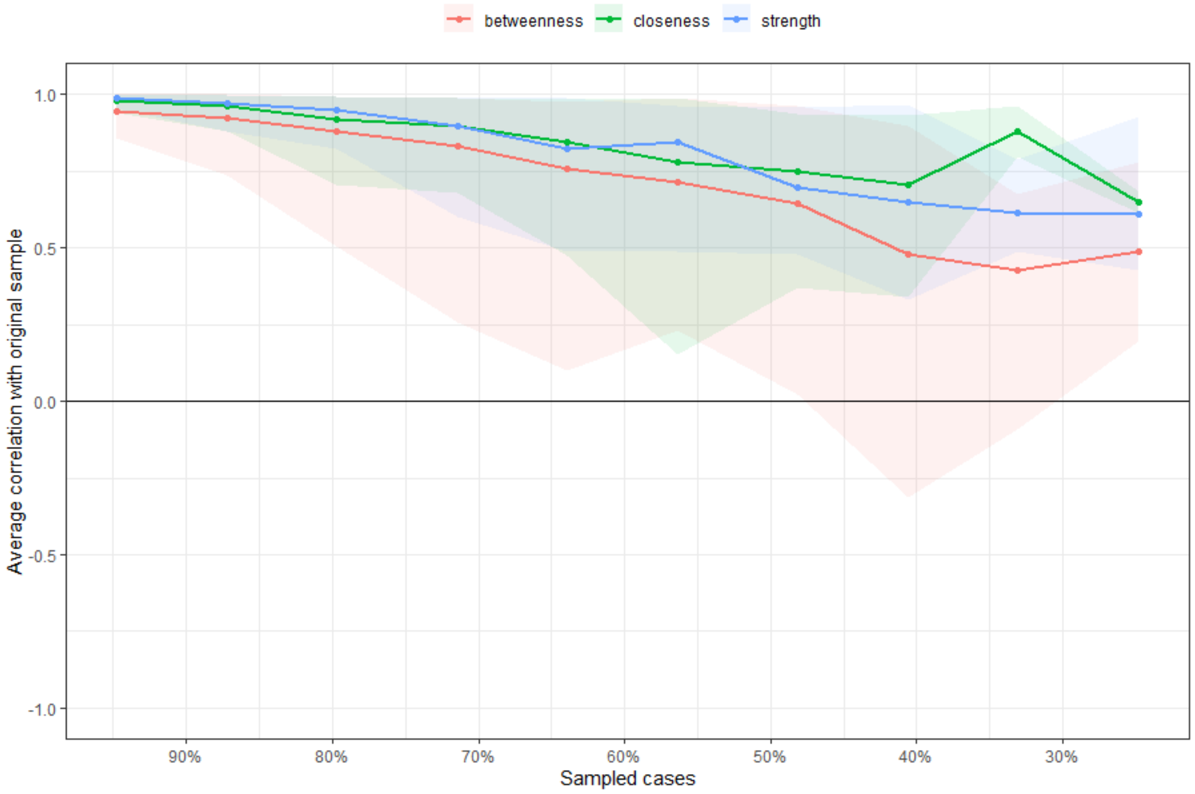

Average correlations between centrality indices of networks sampled with persons dropped and from the original sample. Lines indicate the means and areas indicate the range from the 2.5th quantile to the 97.5th quantile.

E- Anxiety Disorders (N=179)

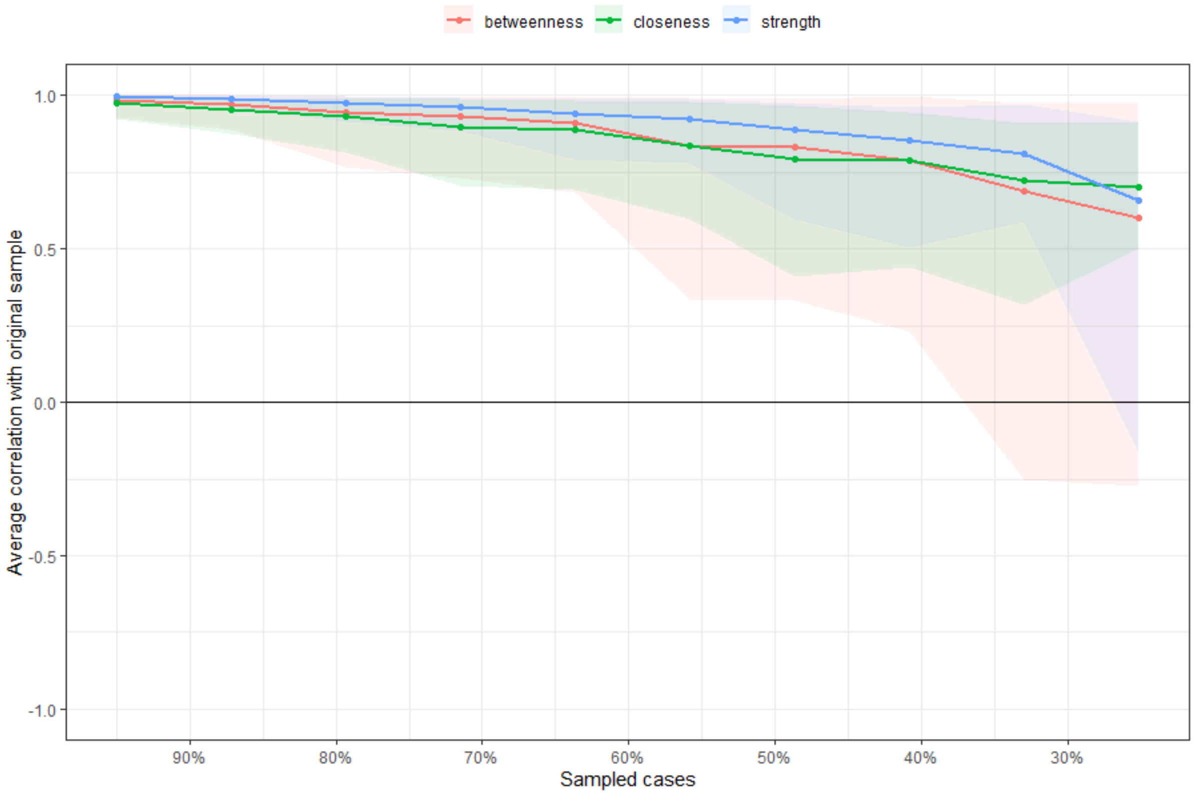

Average correlations between centrality indices of networks sampled with persons dropped and from the original sample. Lines indicate the means and areas indicate the range from the 2.5th quantile to the 97.5th quantile.

F- Personality Disorders (N=230)

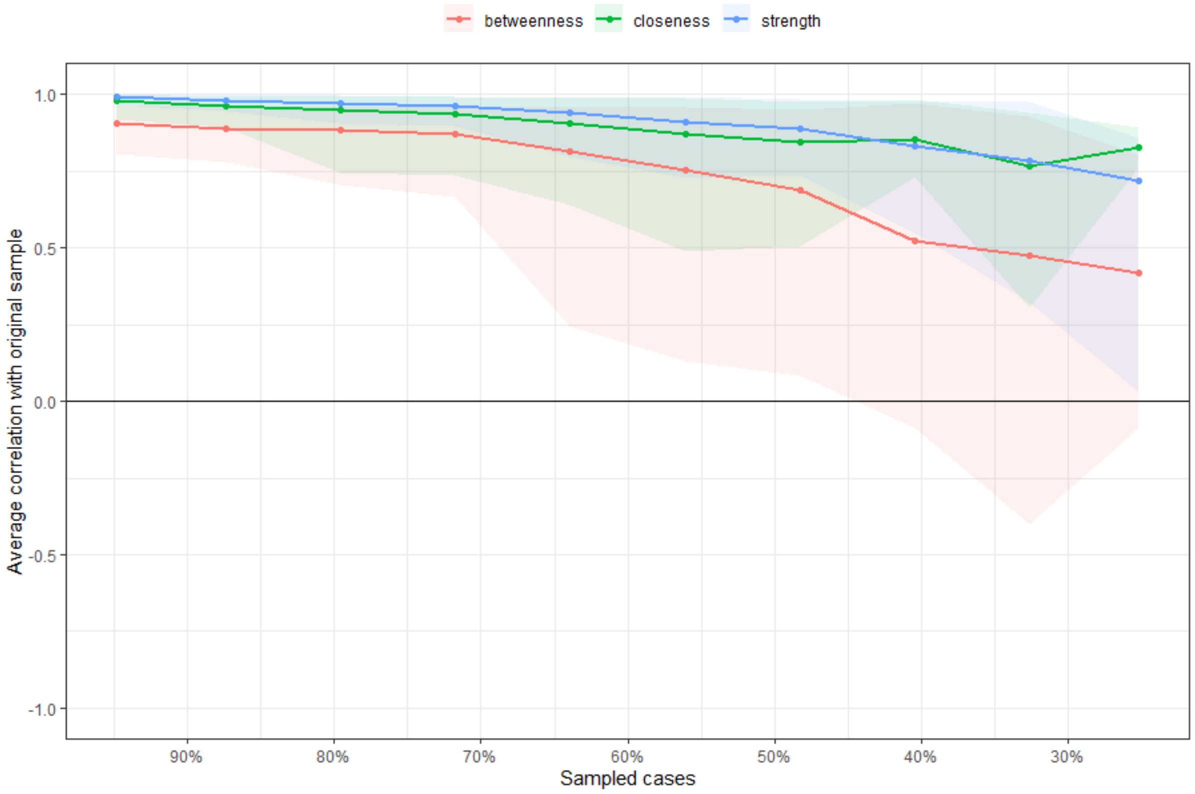

Average correlations between centrality indices of networks sampled with persons dropped and from the original sample. Lines indicate the means and areas indicate the range from the 2.5th quantile to the 97.5th quantile.
