# Supplementary material for: The central role of self-esteem in the quality of life of patients with mental disorders
Source: Sci Rep. 2022 May 12;12:7852. doi: 10.1038/s41598-022-11655-1 (PMC9098638; doi:10.1038/s41598-022-11655-1)
Supplement: Supplementary file 5 — Supplementary Information 5. [file 41598_2022_11655_MOESM5_ESM.pdf]

Supplementary Materials 5. Bootstrapped difference tests (α = 0.05) between node strength for the eight quality-of-life dimensions.

A- Schizophrenia Spectrum Disorders (N=929)

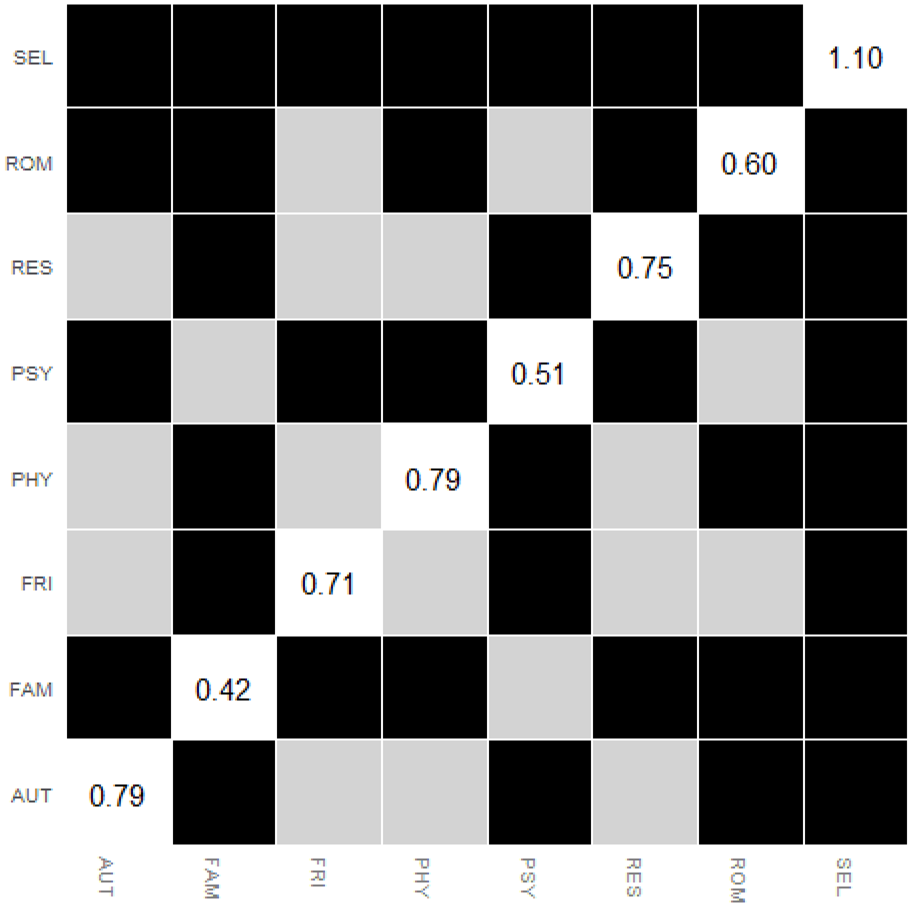

Grey boxes indicate nodes that do not differ significantly from one-another and black boxes represent nodes that do differ significantly from one-another. White boxes show the value of node strength. It is important to note that no correction for multiple testing was applied. Quality-of-life dimensions: self-esteem (SEL), romantic life (ROM), resilience (RES), psychological well-being (PSY), physical well-being (PHY), relationships with friends (FRI), family relationships (FAM), autonomy (AUT).

B- Neurodevelopmental Disorders (N=216)

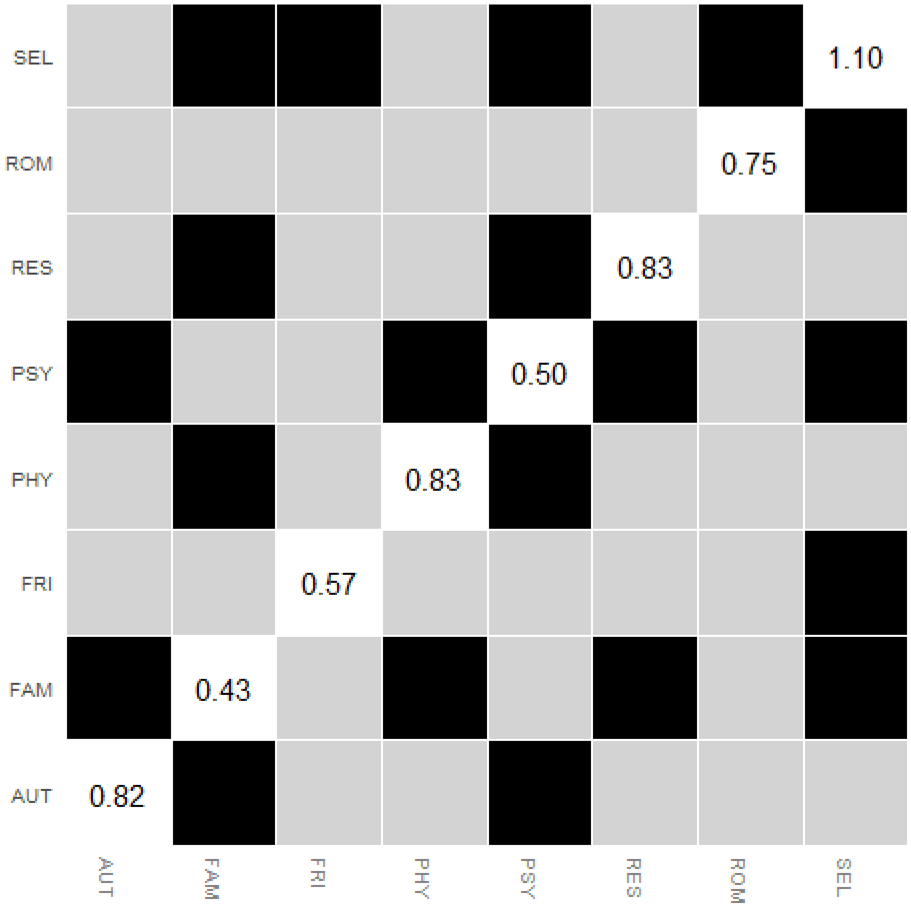

Grey boxes indicate nodes that do not differ significantly from one-another and black boxes represent nodes that do differ significantly from one-another. White boxes show the value of node strength. It is important to note that no correction for multiple testing was applied. Quality-of-life dimensions: self-esteem (SEL), romantic life (ROM), resilience (RES), psychological well-being (PSY), physical well-being (PHY), relationships with friends (FRI), family relationships (FAM), autonomy (AUT).

C- Bipolar Disorders (N=275)

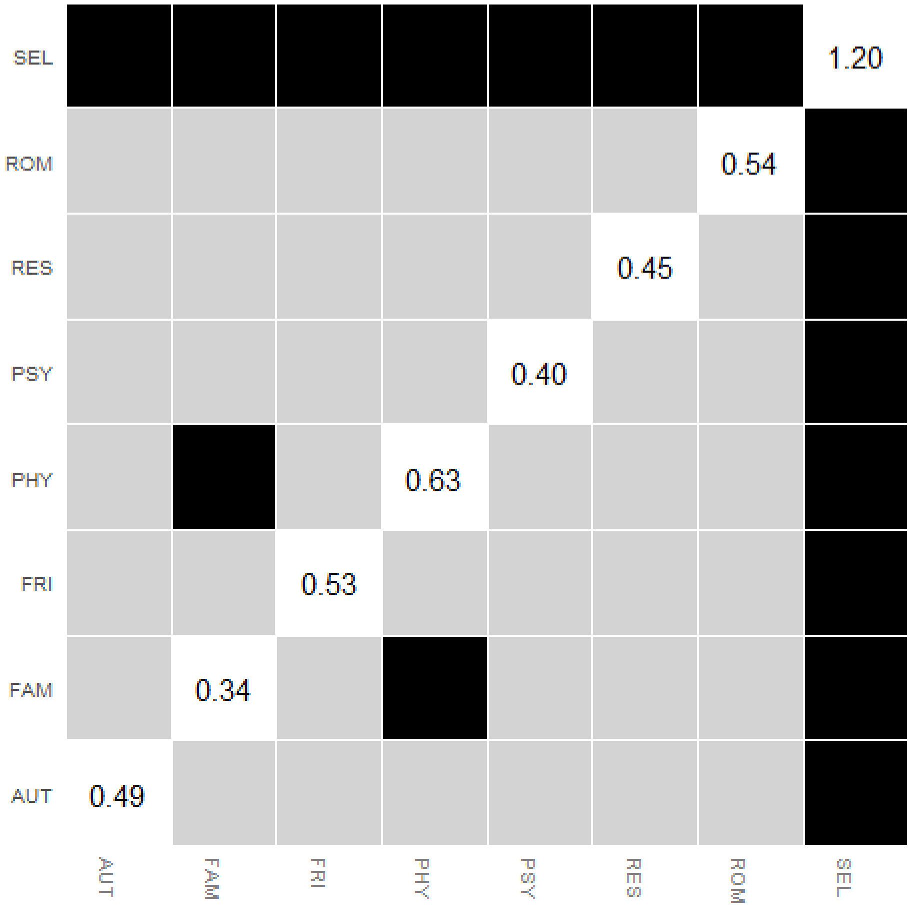

Grey boxes indicate nodes that do not differ significantly from one-another and black boxes represent nodes that do differ significantly from one-another. White boxes show the value of node strength. It is important to note that no correction for multiple testing was applied. Quality-of-life dimensions: self-esteem (SEL), romantic life (ROM), resilience (RES), psychological well-being (PSY), physical well-being (PHY), relationships with friends (FRI), family relationships (FAM), autonomy (AUT).

D- Depressive Disorders (N=133)

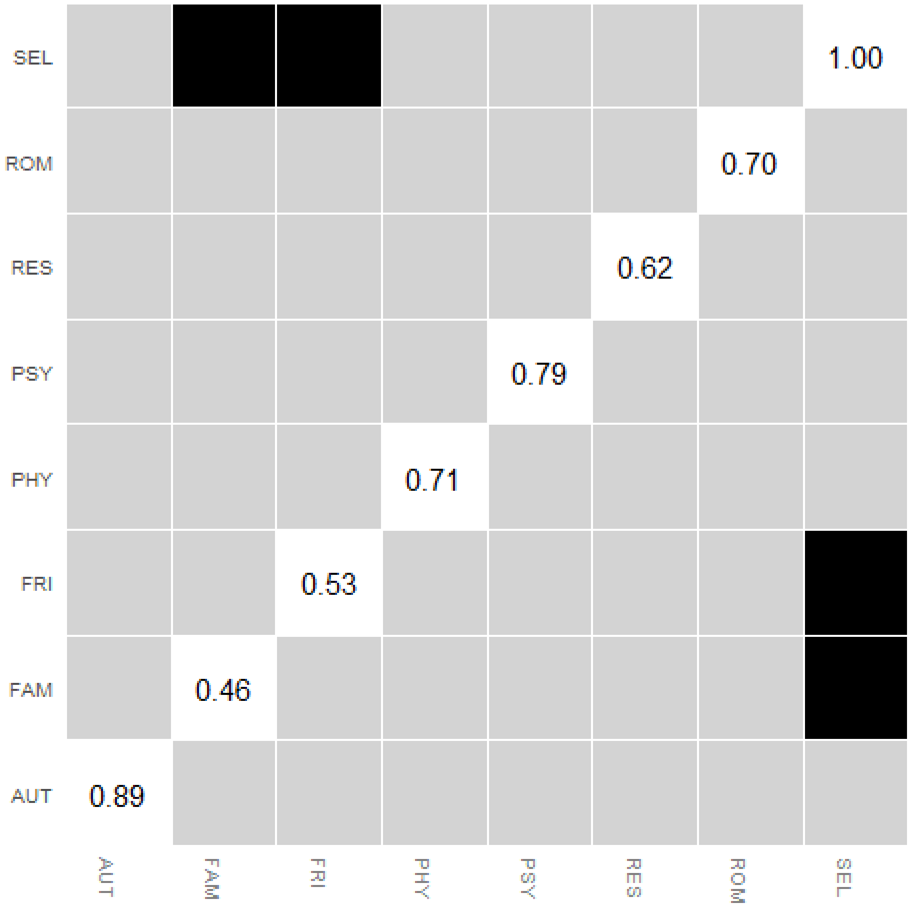

Grey boxes indicate nodes that do not differ significantly from one-another and black boxes represent nodes that do differ significantly from one-another. White boxes show the value of node strength. It is important to note that no correction for multiple testing was applied. Quality-of-life dimensions: self-esteem (SEL), romantic life (ROM), resilience (RES), psychological well-being (PSY), physical well-being (PHY), relationships with friends (FRI), family relationships (FAM), autonomy (AUT).

E- Anxiety Disorders (N=179)

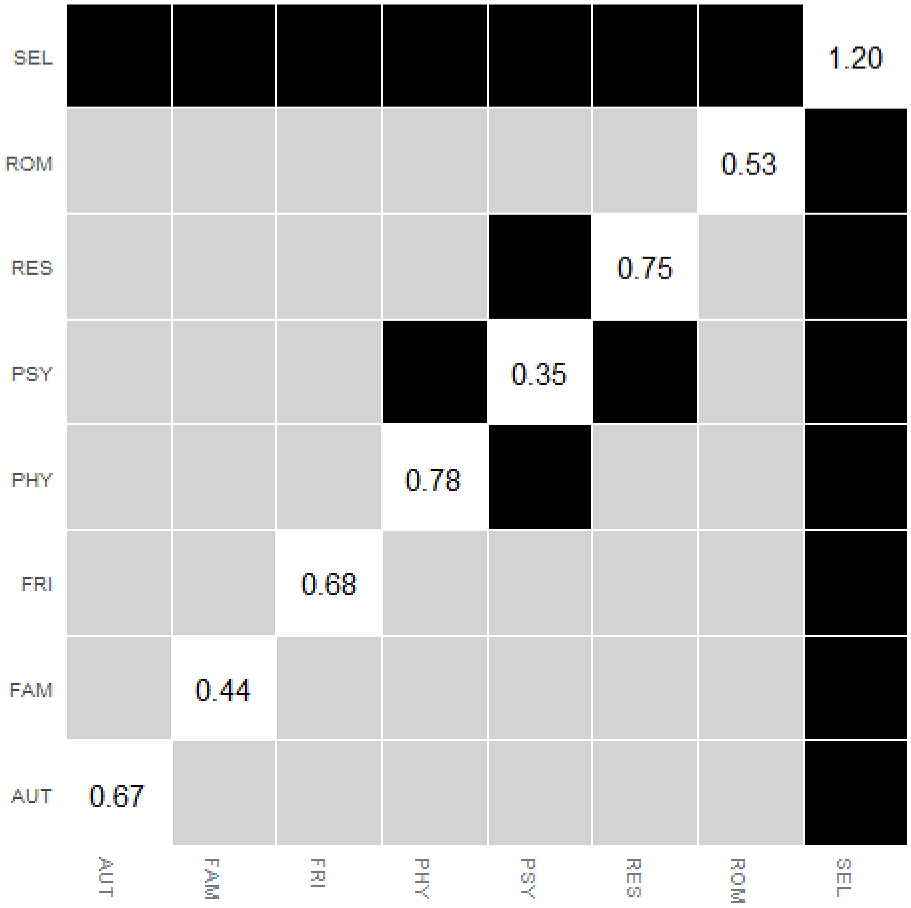

Grey boxes indicate nodes that do not differ significantly from one-another and black boxes represent nodes that do differ significantly from one-another. White boxes show the value of node strength. It is important to note that no correction for multiple testing was applied. Quality-of-life dimensions: self-esteem (SEL), romantic life (ROM), resilience (RES), psychological well-being (PSY), physical well-being (PHY), relationships with friends (FRI), family relationships (FAM), autonomy (AUT).

F- Personality Disorders (N=230)

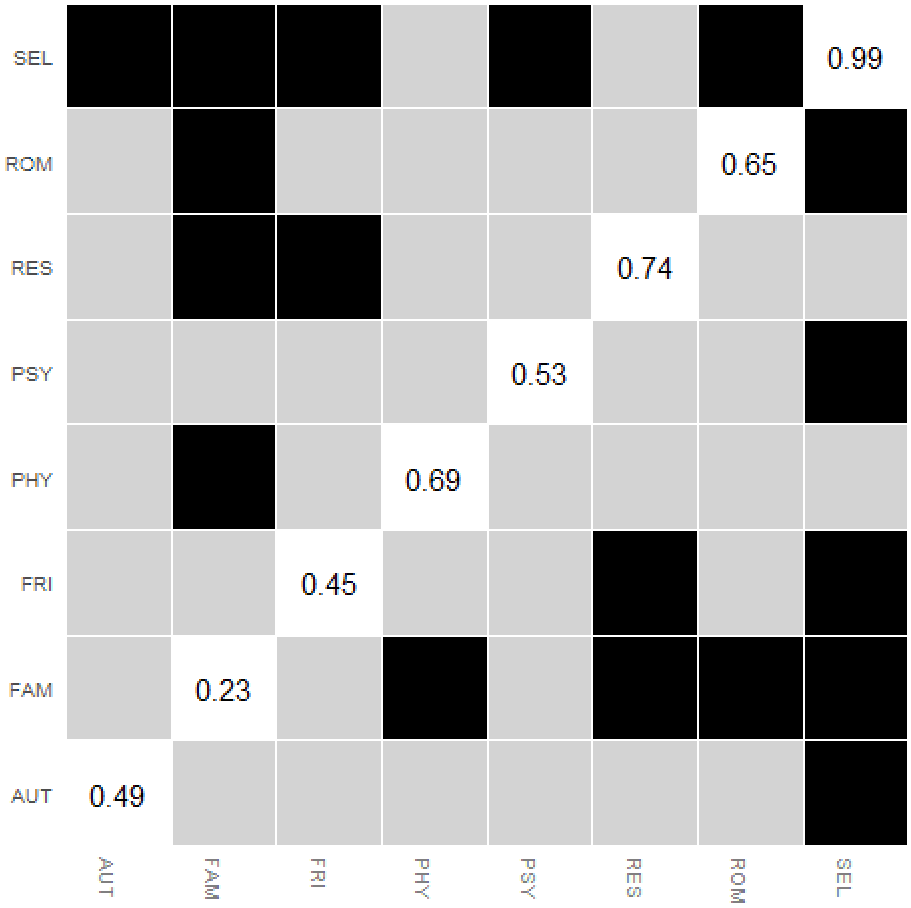

Grey boxes indicate nodes that do not differ significantly from one-another and black boxes represent nodes that do differ significantly from one-another. White boxes show the value of node strength. It is important to note that no correction for multiple testing was applied. Quality-of-life dimensions: self-esteem (SEL), romantic life (ROM), resilience (RES), psychological well-being (PSY), physical well-being (PHY), relationships with friends (FRI), family relationships (FAM), autonomy (AUT).
