# Supplementary material for: The central role of self-esteem in the quality of life of patients with mental disorders
Source: Sci Rep. 2022 May 12;12:7852. doi: 10.1038/s41598-022-11655-1 (PMC9098638; doi:10.1038/s41598-022-11655-1)
Supplement: Supplementary file 6 — Supplementary Information 6. [file 41598_2022_11655_MOESM6_ESM.pdf]

Supplementary Materials 6. Network Comparison Tests. Between-Group Analysis on Network Structure; Overall Connectivity, Edge-Weight; and Node Strength.

**Diagnosis Groups Comparison**	**Network Structure**	**Overall Connectivity**	**Edge-Weight**	**Node Strength**
Schizophrenia Spectrum Disorders vs. Neurodvpt Disorders	p=0.19	p=0.69	all p’s>0.17	all p’s=1
Schizophrenia Spectrum Disorders vs. Bipolar Disorders	p=0.19	**p=0.03**	all p’s>0.48	all p’s>0.15
Schizophrenia Spectrum Disorders vs. Depressive Disorders	p=0.08	p=0.97	all p’s>0.11	all p’s=1
Schizophrenia Spectrum Disorders vs. Anxiety Disorders	p=0.62	p=0.41	all p’s=1	all p’s=1
Schizophrenia Spectrum Disorders vs. Personality Disorders	p=0.44	p=0.10	all p’s>0.48	all p’s>0.45
Neurodvpt Disorders vs. Bipolar Disorders	p=0.13	**p=0.05**	all p’s>0.22	all p’s>0.27
Neurodvpt Disorders vs. Depressive	p=0.12	p=0.74	all p’s>0.22	all p’s=1

Disorders				
Neurodvpt Disorders vs. Anxiety Disorders	p=0.20	p=0.20	all p's>0.28	all p's=1
Neurodvpt Disorders vs. Personality Disorders	p=0.74	p=0.15	all p's>0.20	all p's>0.67
Bipolar Disorders vs. Depressive Disorders	p=0.22	p=0.31	all p's>0.19<br><br>**except AUT--PSY p=0.03**	all p's=1
Bipolar Disorders vs. Anxiety Disorders	p=0.55	p=0.18	all p's=1	all p's>0.98
Bipolar Disorders vs. Personality Disorders	p=0.61	p=0.77	all p's=1	all p's>0.96
Depressive Disorders vs. Anxiety Disorders	p=0.28	p=0.59	all p's>0.34	all p's>0.74
Depressive Disorders vs. Personality Disorders	p=0.31	p=0.61	all p's=1<br><br>**except AUT--FAM p=0.03**	all p's=1
Anxiety Disorders vs. Personality Disorders	p=0.67	p=0.54	all p's>0.22	all p's=1
